# Single‐microbe RNA sequencing uncovers unexplored specialized metabolic functions of keystone species in the human gut

**DOI:** 10.1002/imt2.70035

**Published:** 2025-04-17

**Authors:** Yifei Shen, Wenxin Qu, Mengdi Song, Tianyu Zhang, Chang Liu, Xiaofeng Shi, Xinxin Xu, Jingjing Jiang, Liguo Ding, Fangyu Mo, Zheying Mao, Mingzhu Huang, Ziye Xu, Jiaye Chen, Enhui Shen, Jian Ruan, Jiong Liu, Michael P. Timko, Yu Chen, Longjiang Fan, Shufa Zheng, Yongcheng Wang

**Affiliations:** ^1^ Department of Laboratory Medicine of The First Affiliated Hospital & Liangzhu Laboratory Zhejiang University School of Medicine Hangzhou China; ^2^ Key Laboratory of Clinical In Vitro Diagnostic Techniques of Zhejiang Province Hangzhou China; ^3^ M20 Genomics Hangzhou China; ^4^ Institute of Bioinformatics Zhejiang University Hangzhou China; ^5^ Department of Medical Oncology of The First Affiliated Hospital Zhejiang University School of Medicine Hangzhou China; ^6^ Departments of Biology and Public Health Sciences University of Virginia Charlottesville Virginia USA

**Keywords:** diurnal dynamic, gut microbiome, metabolism, single‐microbe RNA sequencing, species functional heterogeneity

## Abstract

The human body is inhabited by trillions of microorganisms that play a crucial role in health and diseases. Our understanding of the species and functional composition of the human gut microbiome is rapidly expanding, but it is still mainly based on taxonomic profiles or gene abundance measurements. As such, little is known about the species–function heterogeneity and dynamic activities in human microecosystem niches. By applying a novel gut‐specific single‐microbe ribonucleic acid (RNA) sequencing and analytical framework on three healthy donors with distinct enterotypes, we created a comprehensive transcriptional landscape of the human gut microbiome and dissected functional specialization in 38,922 single microbes across 198 species. We investigated the functional redundancy and complementarity involved in short‐chain fatty acids related central carbon metabolism and studied the heterogeneity and covariation of single‐microbe metabolic capacity. Comparing the human gut microbiome at different times throughout the day, we were able to map diurnal dynamic activities of the gut microbiome and discovered its association with sub‐population functional heterogeneous. Remarkably, using single‐microbe RNA sequencing, we systematically dissected the metabolic function heterogeneity of *Megamonas funiformis*, a keystone species in Asian populations. Together with in vitro and in vivo experimental validations, we proved *M. funiformis* can effectively improve mineral absorption through exogenous phytic acid degradation, which could potentially serve as a probiotic that reduces malnutrition caused by deficiency of mineral elements. Our results indicated that species‐function heterogeneity widely exists and plays important roles in the human gut microbiome, and through single‐microbe RNA sequencing, we have been able to capture the transcriptional activity variances and identify keystone species with specialized metabolic functions of possible biological and clinical importance.

## Highlights

- Apply a novel gut‐specific single‐microbe RNA sequencing and analytical framework on three healthy donors with distinct enterotypes.
- Identified diurnal dynamic activities and metabolic heterogeneity involved in central carbon metabolism in the gut microbiome.
- Demonstrated *Megamonas funiformis*, a keystone species in Asian populations, can effectively improve mineral absorption through exogenous phytic acid degradation.

## Graphical abstract

The human body is inhabited by trillions of microorganisms that play a crucial role in health and diseases. Although our understanding of the species and functional composition of the human gut microbiome is rapidly expanding, species–function heterogeneity and dynamic activities in human microecosystem niches remain unclear. By applying a novel gut‐specific single‐microbe RNA sequencing and analytical framework on three healthy donors with distinct enterotypes, we created a comprehensive transcriptional landscape of human gut microbiome. We investigated the functional redundancy and complementarity involved in central carbon metabolism and studied the heterogeneity and covariation of single‐microbe metabolic capacity. We demonstrated *Megamonas funiformis*, a keystone species in Asian populations, can effectively improve mineral absorption through exogenous phytic acid degradation.

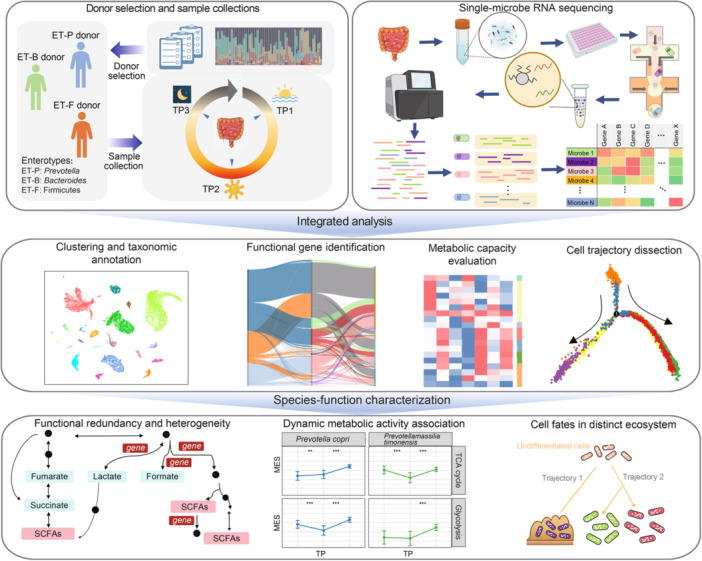

## INTRODUCTION

The human gastrointestinal tract (gut microbiota) consists of a vast number and variety of microbes significantly linked to various health and disease conditions [1]. The gut microbiota plays a crucial role in metabolic processes for the host, including breaking down complex carbohydrates and proteins, and producing essential micronutrients and short‐chain fatty acids (SCFAs) [2]. Functional repartition within the microbiota is influenced by microbial competition, niche specialization, and host‐driven selective pressures towards commensalism and functional redundancy [3]. However, limitations in isolating and cultivating individual colon bacteria have hindered the functional characterization of the gut microbes [4]. Enterotypes are effective in stratifying populations and providing a global overview of the interindividual variations in gut microbial composition [5]. The human gut hosts hundreds of bacterial and archaeal species, with Firmicutes and Bacteroidetes being the dominant phyla [6, 7]. Reproducible patterns of variation in the microbiota, such as the proportions of major taxa like *Bacteroides* and *Prevotella*, have been observed in the adult human gut [8]. As a result, our understanding of functional heterogeneity, ecosystem redundancy and complementarity patterns remains limited. Significant challenges persist in uncovering species‐functional activity heterogeneity and redundancy, impeding groundbreaking insights.

The role of the gut microbiota in regulating circadian rhythms has significant implications for understanding various biological and ecological processes. Circadian rhythms in gut microbiota composition are crucial for metabolic function, yet the extent to which they govern microbial dynamics compared to seasonal and lifetime processes remains unknown [9]. However, our current understanding of the interactions between the gut microbiota and host circadian rhythms relies mainly on changes in relative microbial abundance, and little is known about the diurnal dynamic activities of gut microbiomes. To fully grasp the ecological and evolutionary significance of these interactions, microbiome studies need to be carefully designed with the assistance of novel technologies.

Recent advancements in single‐microbe ribonucleic acid (RNA) sequencing provide opportunities for studying complex microbial communities to assess microbiota functional heterogeneity, niche diversification and adaptative response to host and environmental factors in single microbe resolution [10]. In this study, we applied a combination of a gut‐specific single‐microbe RNA sequencing method and a single‐microbe transcriptional analytical framework: (1) to create a comprehensive transcriptional landscape of the human gut microbiome, and dissected functional specialization in gut species, (2) to investigate functional redundancy and complementarity involved in SCFAs related central carbon metabolism, (3) to study heterogeneity and covariation of single‐microbe metabolic capacity, (4) to map diurnal dynamic activities of functional microbiome throughout the gut ecosystem diversity landscape, and (5) to explore microbe cellular state transitions in distinct colon ecosystems, and identifying the specialized metabolic functions of keystone species in the human gut microbiome.

## RESULTS

### Experimental design and single‐microbe RNA sequencing of the human gut microbiome

Studies on the properties of each enterotype revealed networks of co‐occurring microorganisms centered around one indicator taxon: Enterotype‐*Bacteroides* (ET‐B) enterotype is best indicated by *Bacteroides*; Enterotype‐*Prevotella* (ET‐P) enterotype is driven by *Prevotella*, whose abundance is inversely correlated with *Bacteroides*; and Enterotype‐Firmicutes (ET‐F) enterotype is characterized by an overrepresentation of Firmicutes [8].

**Figure 1:**
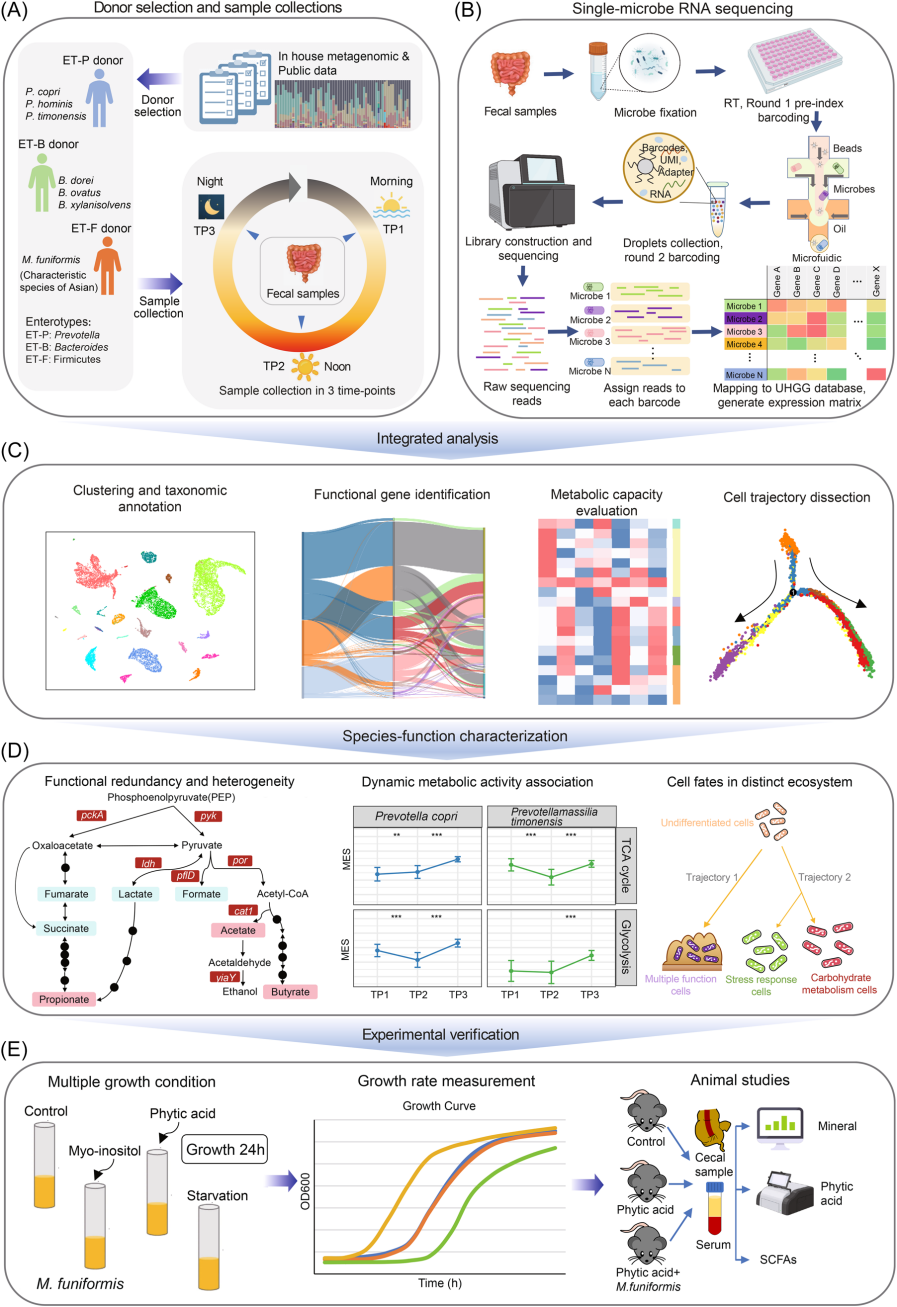
Overview of the experiment design and single‐microbe ribonucleic acid (RNA) sequencing of the human gut microbiome. (A) Overview of the donor selection and sample collections in this study. (B) Workflow of single‐microbe RNA sequencing in human gut microbiome. (C) Integrated analysis framework of single‐microbe RNA sequencing data. (D) Characterized species‐function heterogeneity, diurnal dynamic activities and microbe cellular fates transitions. (E) Experimental verification of the key specialized function species (*Megamonas funiformis*). ET‐B, Enterotype‐*Bacteroides*; ET‐F, Enterotype‐Firmicutes; ET‐P, Enterotype‐*Prevotella*; SCFA, short‐chain fatty acids; TP1, Time point 1; TP2, Time Point 2; TP3, Time point 3.

To systematically investigate the transcriptional activity of the human gut microbiome, we included three healthy donors with representative enterotypes (ET‐P, ET‐B, ET‐F) based on metagenomic analysis from our in‐house clinical cohort [11] (Figure 1A). The ET‐P donor was dominated by the *Prevotella* genus, including well‐studied species such as *P. copri* [12], *P. timonensis* [13], and *P. hominis* [14]. The ET‐B donor was driven by the *Bacteroides* genus, including species like *B. dorei* [15], *B. xylanisolvens* [16], and *B. ovatus* [17]. In the ET‐F donor, the most abundant species was *Megamonas funiformis* [18], a Firmicutes member. Notably, *Megamonas* has not previously been reported as a dominant genus in studies involving European and American subjects but has been found in Chinese and Japanese populations [19], suggesting it may be characteristic of Asian populations. Although several studies have linked *M. funiformis* to human health [19, 20], its function within the human gut microbiome remains poorly understood. To further explore the diurnal dynamics of the human gut microbiome, we collected fecal samples from each donor at three different times during the day (morning: Time point1 (TP1), noon: Time point2 (TP2), night: Time point3 (TP3)) (Figure 1A). All samples were collected during the same 24‐h time period. We then performed single‐microbe RNA sequencing (Figure 1B) (see Methods) and integrated data analysis (Figure 1C) to uncover species‐function heterogeneity and diurnal dynamic activities within the human gut microbiome (Figure 1D), together with the experimental verification (Figure 1E).

In total, we captured over 100,000 gut microbes among all the samples and retained 38,922 high‐quality microbes following quality control (see Methods). We mapped the sequencing reads obtained to the reference genome of the human gut microbiome from the Unified Human Gastrointestinal Genome (UHGG) database [21], ensuring unique alignment to the reference genomes, and quantified the single‐microbe gene expression level in each sample (see Methods). For each donor, we captured a median of over 100 genes, with a total of 2660 genes detected among all the samples (Figure S1A).

### Single‐microbe transcriptional landscape reveals species‐specific functional characterizations in the human gut

**Figure 2:**
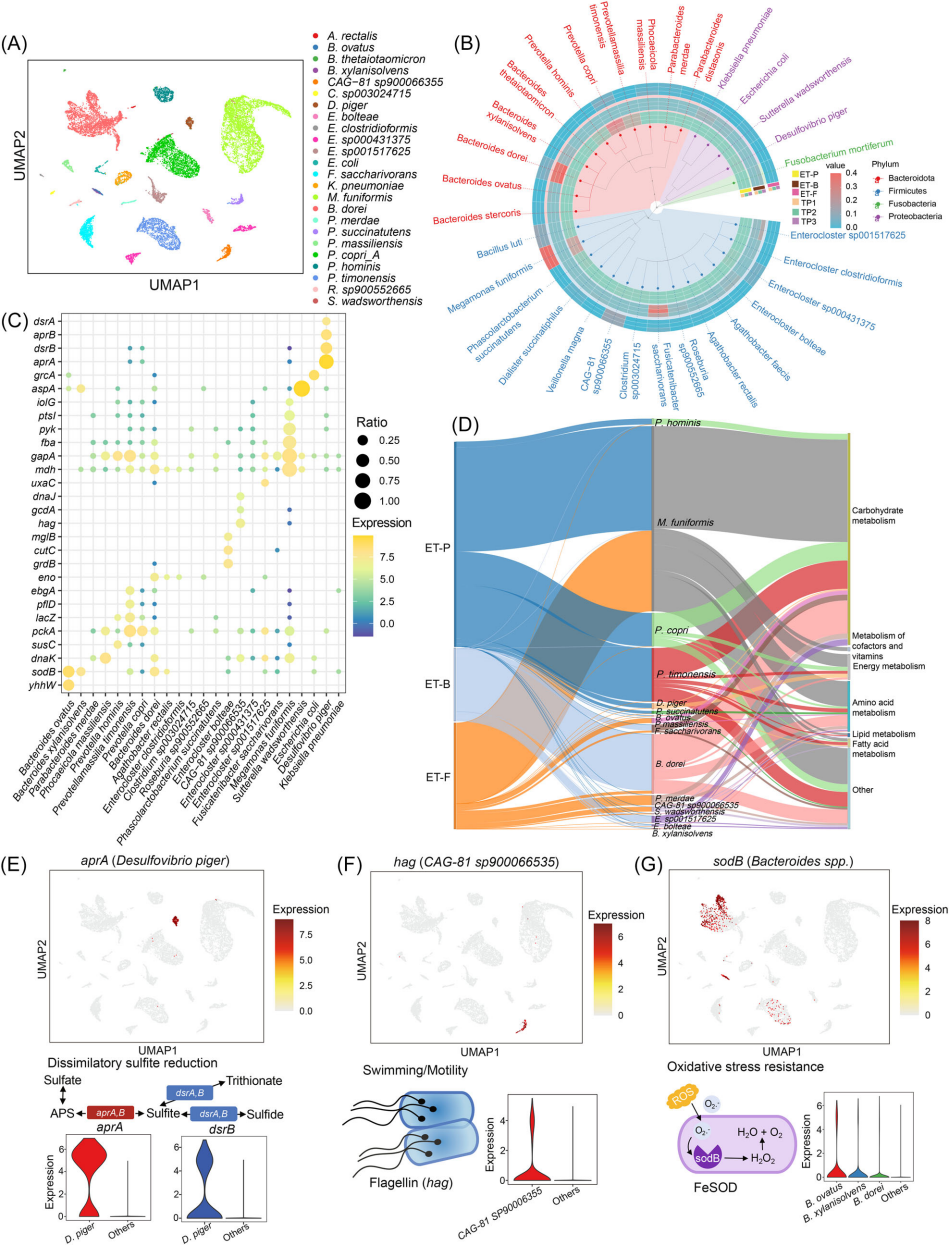
Single‐microbe transcriptional landscape of the human gut and the species‐specific functional characterizations. (A) Uniform manifold approximation and projection (UMAP) of the gut microbes with taxonomic annotation colored by species. (B) Phylogenetic tree plot showing the taxonomic proportion of the gut microbes from nine samples in the three donors. (C) Dot plot showing the functional marker genes in each species. (D) Sankey plot showing the function of species‐specific marker genes in three donors. (E) UMAP color by the gene *aprA* (marker of *Desulfovibrio piger*) expression level, violin plot showed the expression level of genes (*aprA* and *dsrB*) in sulfur metabolism, the pathway diagram illustrates the function of the genes in the “Dissimilatory sulfite reduction.” (F) UMAP color by the gene *hag* (marker of *CAG‐81 sp900066535*) expression level, violin plot showed the expression level of gene *hag* (flagellin), which related with microbe motility. (G) UMAP color by the gene *sodB* (marker of *Bacteroides dorei*) expression level, violin plot showed the expression level of gene *sodB* (superoxide dismutase), which catalyzes the conversion of superoxide radicals to oxygen and hydrogen peroxide, protecting cells from the toxic byproducts of aerobic respiration. ET‐B, Enterotype‐*Bacteroides*; ET‐F, Enterotype‐Firmicutes; ET‐P, Enterotype‐*Prevotella*; FeSOD, iron superoxide dismutase; ROS, reactive oxygen species; TP1, Time point 1; TP2, Time Point 2; TP3, Time point 3; UMAP, uniform manifold approximation and projection.

The human gut microbiome comprises a vast number of microbes with a wide variety of species across different individuals. To annotate single‐microbe taxonomy within this complex ecosystem, we used a *K*‐mer‐based root‐to‐leaf classification strategy, which has proven efficient for identifying microbial species in the human gut microbiome [10]. We identified 198 species among the three donors (Figure S1B) and selected 30 core species (containing more than 100 microbes each) for further functional analysis. Then, we integrated the single‐microbe taxonomic annotations with the uniform manifold approximation and projection (UMAP) [22] dimension reduction clustering results (Figure 2A, S2A). As expected, different clusters corresponded to specific species, with *M. funiformis*, *P. copri*, and *B. dorei* being the most abundant species among the three donors (Figure 2B). These results demonstrate that single‐microbe RNA sequencing effectively captures transcriptional changes, allowing for discrimination of species‐level differences within the human gut microbiome.

Using the transcriptional clusters corresponding to each species, we further analyzed the functional characteristics by identifying species‐specific marker genes (Figure 2C). To compare gene expression levels across different species, we annotated the genes based on Kyoto Encyclopedia of Genes and Genomes functional categories and gene symbols from the UHGG reference genome, and then identified the highly expressed genes in each species. In total, we identified 220 functional marker genes among the 16 species present in the three donors. Among these functional marker genes, 37 (16.8%) were related to amino acid metabolism, 15 (6.8%) to energy metabolism, and 6 (2.7%) to fatty acid metabolism (Figure 2D). Notably, a significant proportion of marker genes (101, 45.9%) were associated with carbohydrate metabolism, indicating functional heterogeneity in carbon metabolism among gut species. These genes were enriched in *M. funiformis*, *P. copri*, *P. timonensis*, and *B. dorei*, indicating functional heterogeneity in carbon metabolism among gut species.

Interestingly, our analysis of the functional marker genes also revealed species‐specific characteristics related to sulfur metabolism, bacterial motility, and stress response (Table S1). For example, *Desulfovibrio piger*, a common sulfate‐reducing bacterium in the human colon [23], significantly expressed two genes, *aprA* and *dsrB*, that are involved in dissimilatory sulfite reduction (Figure 2E, S2B). The genus *CAG‐81*, which is part of the *Lachnospiraceae* family commonly found in healthy humans [24], highly expressed the flagellin gene (*hag*) (Figure 2F), which is linked to bacterial motility and host immune responses. This finding is consistent with previous studies identifying *Lachnospiraceae* as major flagellin producers in the human gut [25]. Additionally, we observed a high expression level of the Superoxide dismutase (*sodB*) gene expression in *Bacteroides* species, including *B. dorei*, *B. xylanisolvens*, and *B. ovatus* (Figure 2G). *Bacteroides*, known for its oxidative stress resistance [26], was reported as benefiting from the high expression levels of *sodB*. In summary, the single‐microbe transcriptional landscape revealed species‐specific functional characteristics and helped uncover specialized function species in the human gut microbiome.

### Functional redundancy and complementarity of SCFAs related central carbon metabolism

**Figure 3:**
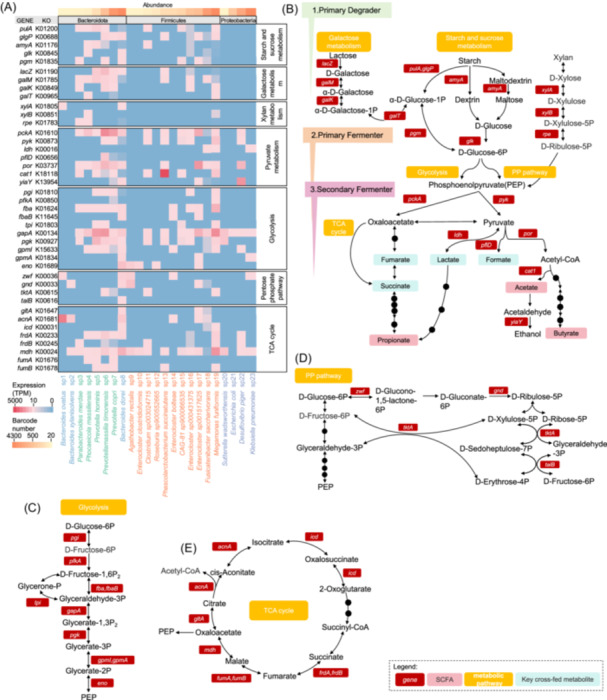
Functional redundancy and complementarity of short‐chain fatty acids (SCFAs) related central carbon metabolism. (A) Heatmap showing the expression level of the genes involved in carbohydrate metabolism. The species within the sample phylum were ordered according to the barcode number. The transcripts per million (TPM) of each species was calculated as following: TPM = (reads/mean detected reads of species) *1,000,000. The pathway diagram shows the galactose metabolism, starch and sucrose metabolism and pyruvate metabolism respectively (B), glycolysis (C), pentose phosphate pathway (PPP) (D) and tricarboxylic acid (TCA) cycle (E). In the context of the human gut microbiome, primary degraders (first trophic level) with specialized machinery hydrolyze complex polysaccharides, releasing sugars accessible to other species. Primary fermenters (second trophic level) either liberate these sugars or acquire them from other microbes, funneling them through glycolysis to produce phosphoenolpyruvate (PEP), which is used for substrate‐level phosphorylation to generate organic acids (e.g., formate, acetate, succinate) or alcohols. Secondary fermenters (third trophic level) use these by‐products to produce SCFAs that affect mucosal and systemic immune responses [27]. PP pathway, pentose phosphate pathway; SCFA, short‐chain fatty acids; TCA cycle, tricarboxylic acid cycle; TPM, transcripts per million.

Given the enrichment of carbon utilization genes among the species‐specific functional marker genes (Figure 2D), we conducted a more detailed analysis of central carbon metabolism‐related genes in each species (Figure 3A).

In the first trophic level, we observed peak expression of genes involved in starch and sucrose metabolism, such as *glgP*, *pulA*, and *amyA*, in the *Prevotella* species from the ET‐P donor (Figure 3B, S3). *Prevotella spp*. are known to be enriched in enzymes for the metabolism of various plant polysaccharides [28]. The *lacZ* gene, encoding β‐galactosidase for lactose metabolism, was expressed in seven species from the ET‐P donor, with the highest expression level in *P. timonensis*. Genes involved in galactose metabolism, such as *galM* and *galK*, also showed the highest expression levels in *Prevotella* species from the ET‐P donor. Additionally, genes associated with xylan metabolism, including *xylA*, *xylB*, and *rpe*, were more highly expressed in the *Bacteroidetes* phylum in ET‐B and ET‐F donors. This aligns with the results of previous studies indicating that the predominant xylan‐degrading organisms in the human colon belong to the *Bacteroidetes* [29].

In the second trophic level, we observed that the gene *pgm*, belonging to the phosphohexose mutase family, was highly expressed in *P. timonensis* from the ET‐P donor and in *B. dorei* from the ET‐B donor (Figure 3B, S3). Genes involved in glycolysis showed highest expression in the dominant species of each donor (Figure 3C, S3). The pentose phosphate pathway (PPP), essential for maintaining carbon homeostasis and providing precursors for nucleotide and amino acid biosynthesis [30], also showed notable gene expression patterns (Figure 3D, S3). Specifically, the genes *zwf* and *gnd*, encoding glucose 6‐phosphate dehydrogenase and 6‐phosphogluconate dehydrogenase, exhibited high expression in *B. dorei* and *Agathobacter rectalis* from the ET‐B donor. Further analysis revealed that genes responsible for complex interconversion reactions at the core of the non‐oxidative PPP, such as transketolase (*tktA*) and transaldolase (*talB*), were highly expressed in *P. timonensis* from the ET‐P donor and *B. dorei* from the ET‐B donor. *tktA* and *talB* act as bridges between glycolysis and the PPP by sharing intermediate metabolites (fructose‐6‐phosphate and glyceraldehyde‐3‐phosphate) [30]. In glycolysis, phosphoenolpyruvate (PEP) generated by glycerate‐2P and catalyzed by enolase forms a crucial node with pyruvate and oxaloacetate, which lies at the junction between glycolysis and the tricarboxylic acid (TCA) cycle, as well as other metabolic pathways [31].

In the third trophic level, phosphoenolpyruvate carboxykinase (*pckA*) and pyruvate kinase (*pyk*) catalyze the conversion of PEP to oxaloacetate and pyruvate, respectively. We observed that *pckA* was most highly expressed in *P. timonensis* from the ET‐P donor and in *Enterocloster sp001517625* from the ET‐B donor, while *pyk* showed the highest expression in *M. funiformis* from the ET‐P and ET‐F donors (Figure 3C, S3). Oxaloacetate is a key component of the TCA cycle. Genes associated with the production of oxaloacetate, namely citrate synthase (*gltA*), aconitate hydratase (*acnA*), and isocitrate dehydrogenase (*icd*), were most highly expressed in *Bacteroides spp*. from the ET‐B donor (Figure 3E, S3). Genes involved in succinate dehydrogenase, such as *frdA* and *frdB*, exhibited the highest expression in *P. timonensis* and *M. funiformis* from the ET‐P and ET‐F donors, respectively. Malate dehydrogenase (*mdh*), which catalyzes the reversible oxidation of malate to oxaloacetate [32], was found to have high expression levels in seven species from the ET‐F donor, with the highest in *M. funiformis*. Pyruvate, a central metabolite for the production of various chemicals, is metabolized by l‐lactate dehydrogenase (*ldh*) to generate lactate [33]. The gene *ldh* showed the highest expression in *Fusicatenibacter saccharivorans* from the ET‐B donor. Formate acetyltransferase (*pflD*), which catalyzes pyruvate to formate, was highly expressed in *P. timonensis* from the ET‐P donor. The gene *por*, which converts pyruvate into acetyl coenzyme A (acetyl‐CoA), was expressed in 11 species, with the highest expression in *E. sp001517625* from the ET‐B donor. Notably, the gene *cat1*, which catalyzes CoA transferase reactions [34], was highly expressed in *Phascolarctobacterium succinatutens*. This observation is consistent with the results of previous studies indicating that *P. succinatutens* uses succinate as a substrate rather than carbohydrates for growth in energy‐limited environments, a strategy for survival in the human gut [10, 34]. In summary, single‐microbe RNA sequencing revealed functional redundancy and complementarity patterns in central carbon metabolism among distinct bacterial species in the human gut microbiome.

### Heterogeneity and covariations of single‐microbe metabolic functions in the human gut

**Figure 4:**
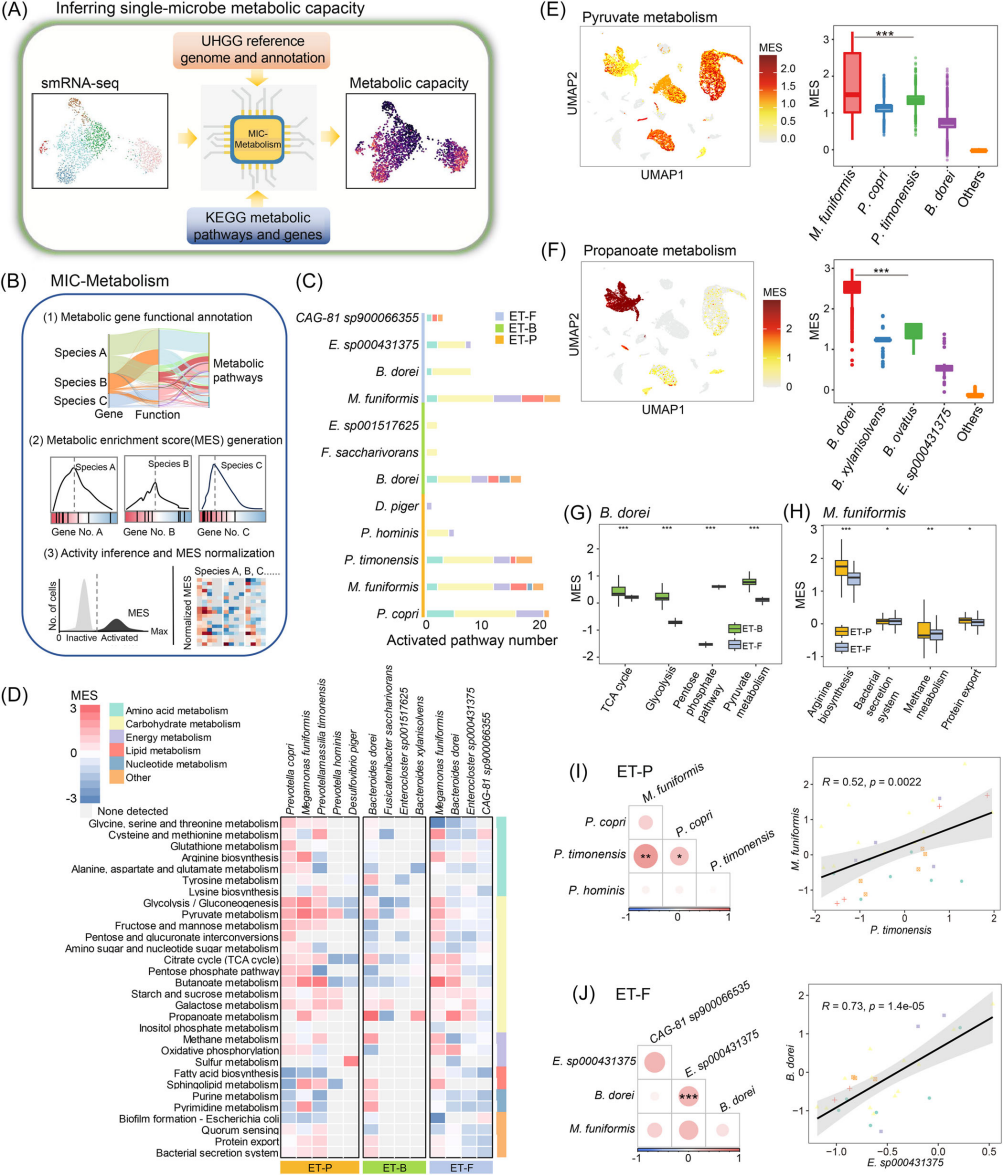
Heterogeneity and covariations of single‐microbe metabolic functions in the human gut. (A) A brief introduction to Microbe‐Metabolism (MIC‐Metabolism). (B) Computational workflow of MIC‐Metabolism. (C) The activated pathway number of the dominant species from the three donors. (D) Heatmap showing the ranked‐based metabolic enrichment score (MES) of the gut microbe species in metabolic‐related pathways. (E) The UMAP colored by the MES of pyruvate metabolism, the boxplot shows the comparison of MES between *M. funiformis* and other microbe species. (F) The UMAP colored by the MES of propanoate metabolism, the boxplot shows the comparison of MES between *B. dorei* and other microbe species. The boxplot shows the comparisons of MES of *B. dorei* (G) and *M. funiformis* (H) in different donors. Correlation analysis of MES for the dominant species of ET‐P donor (I) and ET‐F donor (J), the correlation between *M. funiformis* and *P. timonensis* (I), *B. dorei* and *Enterocloster sp000431375* (J), the dots were colored by the function category of the pathways. *p < 0.05, **p < 0.01, ***p < 0.001. ET‐B, Enterotype‐*Bacteroides*; ET‐F, Enterotype‐Firmicutes; ET‐P, Enterotype‐*Prevotella*; smRNA‐seq, single‐microbe RNA sequencing; UMAP, uniform manifold approximation and projection.

Given the changes in metabolism‐related genes observed in our single microbe analysis (Figure 2D), to systematically dissect the metabolic capacity heterogeneity, we conducted a single‐microbe metabolic gene enrichment analysis for each species (Figure 4A). We developed Microbe‐Metabolism (MIC‐Metabolism) for this purpose (Figure 4B), which involves three steps: (1) metabolic gene functional annotation, (2) ranked‐based metabolic enrichment score (MES) generation, and (3) permutation‐based MES normalization and activity inference for each species (for additional details see Methods). Then, we applied MIC‐Metabolism on the single‐microbe RNA sequencing data from three donors to evaluate metabolic pathway activities in each species (Figure 4C,D).

In the ET‐P donor, we observed peak MES for pathways involved in glycolysis and pyruvate metabolism in *Prevotella spp*. (*P. copri* and *P. timonensis*) and *M. funiformis* (Figure 4D). This is consistent with our findings in Figure 3, suggesting these species dominate carbon metabolism in the gut microbiome. Additionally, we found significant activation of the sulfur metabolism pathway in *D. piger*, consistent with our results shown in Figure 2E. For the ET‐F donor, we noted peak MES for pathways involved in the TCA cycle, glycolysis, and pyruvate metabolism in *M. funiformis* (Figure 4E), as well as peak MES for propanoate metabolism in *B. dorei*. In the ET‐B donor, peak MES was observed for pathways involved in propanoate metabolism in *B. dorei*, and further analysis indicated activity in other *Bacteroides* species as well (Figure 4F). This supports previous studies identifying *Bacteroides* as a dominant group in propanoate metabolism [35]. In summary, these Asian population findings indicate that single‐microbe RNA sequencing combined with MIC‐Metabolism effectively captures the metabolic activity of each species in the human gut microbiome.

Given the presence of *B. dorei* and *M. funiformis* in different enterotype donors, we conducted a comparative analysis to investigate the metabolic capacity of these species across donors. Pathways such as glycolysis, pyruvate metabolism, and the TCA cycle showed significantly lower activity in the ET‐F donor compared to the ET‐B donor (Figure 4G). *M. funiformis* exhibited peak MES for pathways related to carbon metabolism in both donors. However, in the ET‐P donor, the MES for bacterial secretion systems, protein export, and arginine biosynthesis pathways were significantly higher compared to the ET‐F donor. Conversely, pathways related to energy metabolism, such as oxidative metabolism, had higher MES in the ET‐F donor (Figure 4H). These results indicate metabolic capacity heterogeneity of gut species across different human gut microbiomes.

To investigate the activity of metabolic capacity relationships between different species, we performed a correlation analysis of MES in each donor's gut microbiome. In the ET‐P donor, we found that highly activated carbon metabolism species like *P. timonensis* and *M. funiformis* were significantly co‐activated. In the ET‐F donor, the MES of *B. dorei* was significantly positively correlated with *Enterocloster sp000431375* (Figure 4I,J). Further analysis of different functional metabolic pathways in the ET‐P donor's gut microbiome revealed that *P. timonensis* and *P. copri* were significantly co‐activated in carbohydrate metabolism pathways. Additionally, the MES of *P. timonensis* was significantly positively correlated with *M. funiformis* in lipid metabolism pathways (Figure S4). In energy metabolism pathways, the MES of *P. copri* was significantly positively correlated with *M. funiformis* (Figure S4). These findings suggest that the activity of different metabolic functions can be covaried by multiple coexisting bacteria species in distinct human gut microbiome.

### Diurnal dynamic activities associated with sub‐population functional heterogeneous

**Figure 5:**
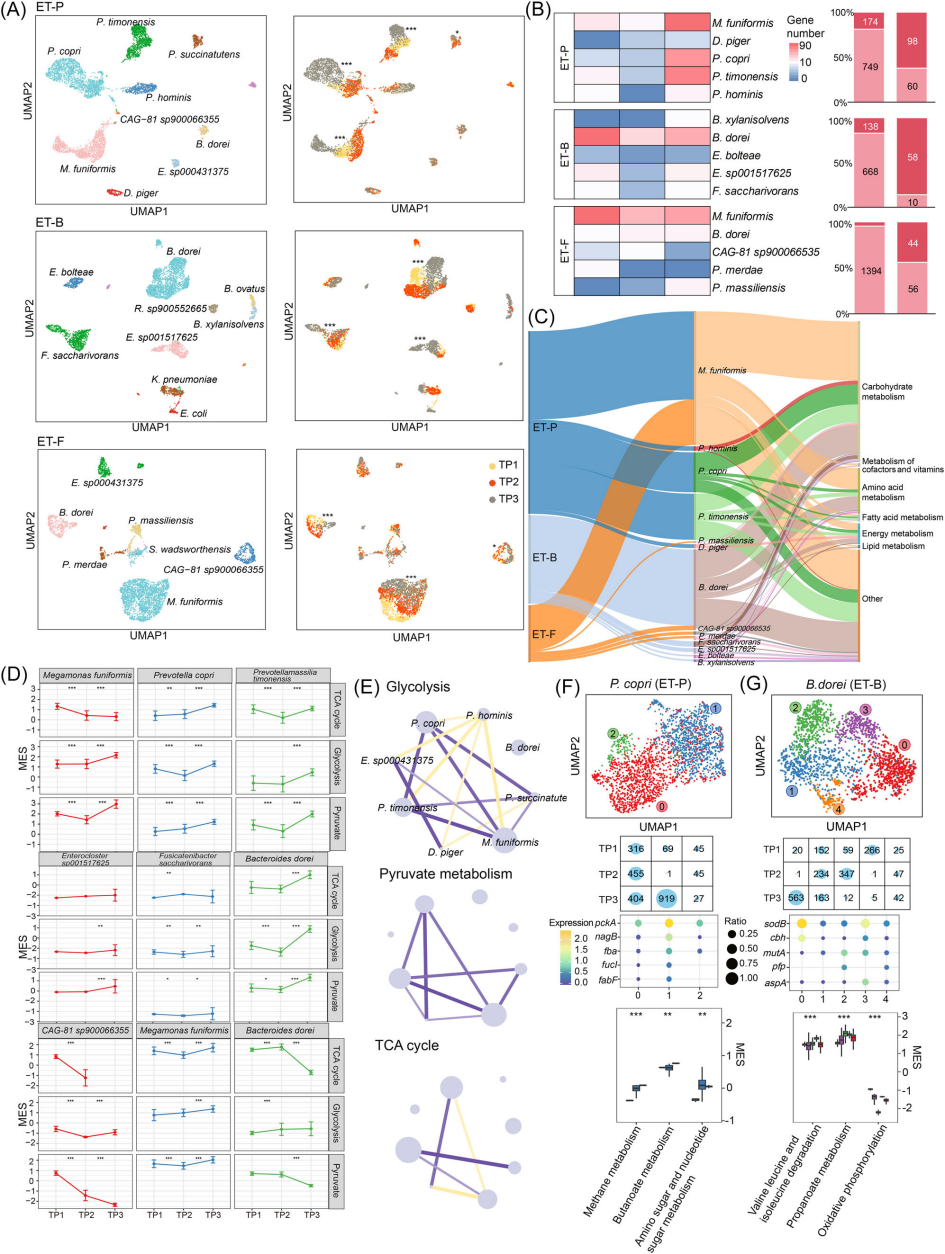
Single‐microbe RNA sequencing captures the diurnal dynamic activities of the human gut microbiome. (A) UMAP color by species (left) and time point (right) of the three donors, the significance was determined by the analysis of similarities (ANOSIM). (B) Heatmap showing the number of time‐point marker gene in the dominant species of the three donors, the bar plot (left) showed the proportion of time‐specific marker genes among all genes of each donor, the bar plot (right) showed the proportion of time marker genes among species marker genes. (C) Sankey plot showing the function of the time‐specific marker genes in the three donors. (D) Comparison of MES on TCA cycle, glycolysis and pyruvate metabolism of different species in the three donors across three‐time points. (E) Correlation of the dominant species from ET‐P donor across different metabolic pathways. The line color purple indicates a positive correlation, while yellow indicates a negative correlation. The thickness of the lines represents the magnitude of the correlation coefficient, and the size of the dots represents the MES of species. (F) UMAP plot showed three clusters (subpopulations) of *P. copri* from ET‐P donor under 0.5 resolution of Seurat package; Dot plot showed the marker genes of each subpopulation; Boxplot showing the MES of specific pathways of each subpopulation; Ballon plot showed the barcode number of each subpopulation and each time point. (G) UMAP plot showing five clusters (sub‐populations) of *B. dorei* from ET‐B donor under 0.5 resolution of Seurat package; Dot plot showing the marker genes of each subpopulation; Boxplot showed the MES of specific pathways of each subpopulation; Ballon plot showing the barcode number of each subpopulation and each time point. *p < 0.05, **p < 0.01, ***p < 0.001. ET‐B, Enterotype‐*Bacteroides*; ET‐F, Enterotype‐Firmicutes; ET‐P, Enterotype‐*Prevotella;* MES, metabolic enrichment score; TCA cycle, tricarboxylic acid cycle; TP1, Time point 1; TP2, Time Point 2; TP3, Time point 3; UMAP, uniform manifold approximation and projection.

To comprehensively examine the dynamic functional activity of the human gut microbiome at single‐microbe resolution, we performed the analysis on three healthy subjects at three different time points within 1 day (Figure 1). Unsupervised clustering analysis of the single‐microbe RNA sequencing datasets from the three donors revealed significant transcriptional alterations between different time points in each species (Figure 5A). To further explore whether the different transcriptional‐level of genes based on time or gene itself, we identified time‐specific marker genes for species displayed in the figure (Figure 5B and Table S2). In those species, we observed a fewer number of marker genes in TP2 among the three donors. Conversely, a significantly higher number of marker genes in these species were present in TP3 in the ET‐P donor. Interestingly, further investigation found the identified time‐specific genes were significantly enriched in the species‐specific marker genes (*p* < 0.05, Chi‐square test) (Figure 5B) among three donors, which suggested that species marker genes contribute more dynamic activities in the human gut microbiome. Additionally, gene functional analysis revealed that most time‐specific marker genes were related to carbohydrate metabolism and amino acid metabolism (Figure 5C).

To further evaluate the diurnal dynamics of metabolic pathway activities in each species, we generated the MES for each pathway using MIC‐metabolism at each time point. Comparing the MES across different time points revealed significant heterogeneity in the dynamic activities of different species within the human gut microbiome (Figure 5D). For example, in the ET‐P donor, the MES for the TCA cycle, glycolysis, and pyruvate metabolism pathways showed similar dynamic patterns in *P. copri*, *P. timonensis*, and *M. funiformis*. In contrast, in the ET‐B donor, only *B. dorei* exhibited dynamic changes in these pathways compared to other species. Additionally, in the ET‐F donor, we observed opposite dynamic patterns between *M. funiformis* and other species like *B. dorei* and *CAG‐81 sp900066535*. We also conducted a MES correlation analysis to investigate pathway activity co‐occurrence patterns between species. Pyruvate metabolism and glycolysis pathways were co‐activated in *P. copri*, *P. timonensis*, and *M. funiformis* in the ET‐P donor. However, in the TCA cycle, there was a negative correlation between *M. funiformis* and *P. copri* (Figure 5E). In summary, our metabolic pathway activity and correlation analyses revealed the complexity of dynamic activity relationships between different species in the human gut microbiome, potentially influenced by circadian rhythms, diet, and microbiome niches.

Since we observed dynamic activity of bacteria in human gut microbiomes, we hypothesize that the diurnal dynamic activities might be caused by the sub‐population functional heterogeneous. To test this hypothesis, we extracted the dominant species, *B. dorei*, and *P. copri* cells, from the ET‐B and ET‐P donors and performed sub‐clustering analysis in each species. Among the three sub‐clusters in the *P. copri*, one subcluster which enriched in TP3 showed significantly higher metabolic activities than others. The genes *fba* and *pckA*, were found to be expressed higher in cluster 1 of *P. copri* (Figure 5F and Table S3). In *B. dorei* of ET‐B donor, we identified five functional clusters of cells based on differentially expressed genes, including oxidative phosphorylation cells (cluster 0), *mutA* ^+^ propanoate metabolism cells (cluster 2) and *aspA* ^+^ alanine, aspartate and glutamate metabolism cells (cluster 3) (Table S3). We also observed that at different time points in ET‐B donor, *B. dorei* exhibited different functional clusters. For example, the cells in functional cluster “oxidative phosphorylation cells” were specifically enriched in TP3, while the functional cluster “*aspA* ^+^ alanine, aspartate and glutamate metabolism cells” were enriched in TP1, and the functional cluster “*mutA* ^+^ propanoate metabolism cells” were enriched in TP2 (Figure 5G). These results showed that the diurnal dynamic activities were associated with the sub‐population functional heterogeneous of the human gut microbiome.

### Cell state transitions of *Megamonas funiformis* in distinct colon ecosystems

**Figure 6:**
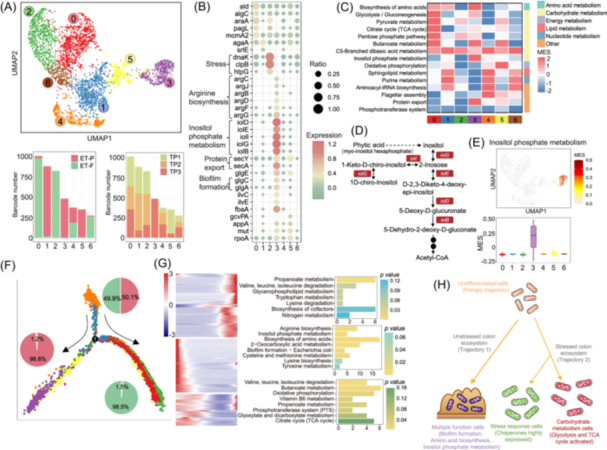
Functional trajectory analysis uncovering *M. funiformis*'s state transitions in distinct colon ecosystems. (A) UMAP clustering identified seven clusters (sub‐populations) of *M. funiformis* from ET‐P and ET‐F donor under 0.5 resolution of Seurat package. Bar plot showed the contributions of different donors (left) and time points (right) of different clusters. (B) Dot plot showed the marker genes of each cluster. (C) Heatmap showing the MES of different pathways across each cluster. (D) Diagram of inositol phosphate metabolism. (E) UMAP colored by MES of inositol phosphate metabolism, the boxplot showing the comparison of MES on inositol phosphate metabolism of each cluster. (F) Pseudo‐time analysis of *M. funiformis*. (G) Differential expressed genes from pseudo‐time analysis. The heatmap shows the time series of gene expression. The bar plot shows the numbers of differential genes per trajectories in the Kyoto Encyclopedia of Genes and Genomes (KEGG) pathways. (H) The underlying mechanism of cell fate transitions within distinct colon ecosystems. ET‐B, Enterotype‐*Bacteroides*; ET‐F, Enterotype‐Firmicutes; ET‐P, Enterotype‐*Prevotella*; MES, metabolic enrichment score; TP1, Time point 1; TP2, Time Point 2; TP3, Time point 3; UMAP, uniform manifold approximation and projection.

*M. funiformis* is a characteristic species in the gut microbiomes of Asian populations [19]. Despite its recognized importance for human health [20], its in situ metabolic patterns and roles within the complex gut microbial community are under‐characterized. Through MIC‐metabolism analysis, we found that *M. funiformis* exhibits metabolic variation across different donors' gut microbial communities (Figure 3). To further investigate the functional heterogeneity of *M. funiformis* cells, we extracted the annotated *M. funiformis* cells from the single‐cell transcriptomes for clustering and functional dissection. Among all the *M. funiformis* cells, we identified seven distinct clusters based on UMAP clustering analysis (Figure 6A), demonstrating that species‐level microbes can be further categorized into different functional sub‐populations. Clustering analysis revealed that clusters 0, 2, and 6 were predominately from the ET‐F donor, clusters 1, 3, and 5 from the ET‐P donor, and cluster 4 was present in both donors (Figure 6A, S5A,B).

Next, we analyzed the functional characteristics of the clusters by identifying marker genes and evaluating metabolic capacity. In cluster 2, which primarily consisted of cells from the ET‐F donor, we found high expression of genes related to protein folding, such as *clpB* and chaperonins (*dnaK* and *htpG*). Cluster 3, mainly from the ET‐P donor, showed significant expression of genes involved in arginine biosynthesis, inositol phosphate metabolism, protein export, and biofilm formation (Figure 6B and Table S3). Given the high number of marker genes in cluster 3, we conducted an enrichment analysis on these genes (Figure S5C). We further calculated and compared the MES across clusters to identify activated pathways. Cluster 0 exhibited the highest MES for central carbon metabolism pathways, including the TCA cycle, glycolysis, and pyruvate metabolism (Figure 6C). In contrast, cluster 3 showed significantly higher MES for inositol phosphate metabolism and protein export compared to other clusters (Figure 6D,E).

To investigate the dynamic transformation of cellular states driven by functional genes, we performed pseudotime analysis [36] on *M. funiformis* cells. This analysis revealed three distinct cell fate trajectories (Figure 6F): undifferentiated cells (primary trajectory, 951 cells), multiple function cells (cell fate 1, 869 cells), and stressed cells (cell fate 2, 2403 cells). Multiple function cells were characterized by specific functional genes, such as those encoding protein translocase subunits (*secY* and *secA*), genes involved in arginine biosynthesis (*argB‐D*, *argF‐G*, *argJ*), and genes involved in inositol phosphate metabolism (*iolB*, *iolD‐E*, *iolG*, *iolI*). Stressed cells were marked by high expression level of chaperonin genes related to intrinsic cellular stresses. The predicted cell metabolic fate trajectories moved from undifferentiated cells to multiple‐function cells and stressed cells.

The pseudotime analysis also revealed 269 genes that co‐vary across the cell state transitions (Figure 6F), with 77 of these genes significantly enriched in two metabolic states involving in eight pathways (Figure 6G). Notably, cells with multiple functions were determined by the Wilcoxon rank‐sum test (*p* < 0.001) to be significantly more active than stressed cells in the “Biofilm formation ‐ *Escherichia coli*” pathway. Enriched genes were primarily concentrated in the “Glycogen biosynthesis” pathway, which is crucial for biofilm formation. This process involves bacteria producing exopolysaccharides and transporting them to the extracellular environment or cell envelope to promote biofilm development [37]. Our pseudotime and enrichment analysis together revealed that the “Glycogen biosynthesis” process plays an essential role in the functional cluster transformation of *M. funiformis*, where undifferentiated cells transition into multiple function cells and stressed cells (Figure 6H). Interestingly, stressed cells exhibited significant heterogeneity in metabolic activity. Carbohydrate metabolism cells were notably more active than stress response cells in central carbon metabolism. Taken together, these results offer new insights into microbe functional heterogeneity within distinct gut microbial ecosystems at the single‐microbe resolution.

### *M. funiformis* improve mineral absorption through exogenous phytic acid degradation

**Figure 7:**
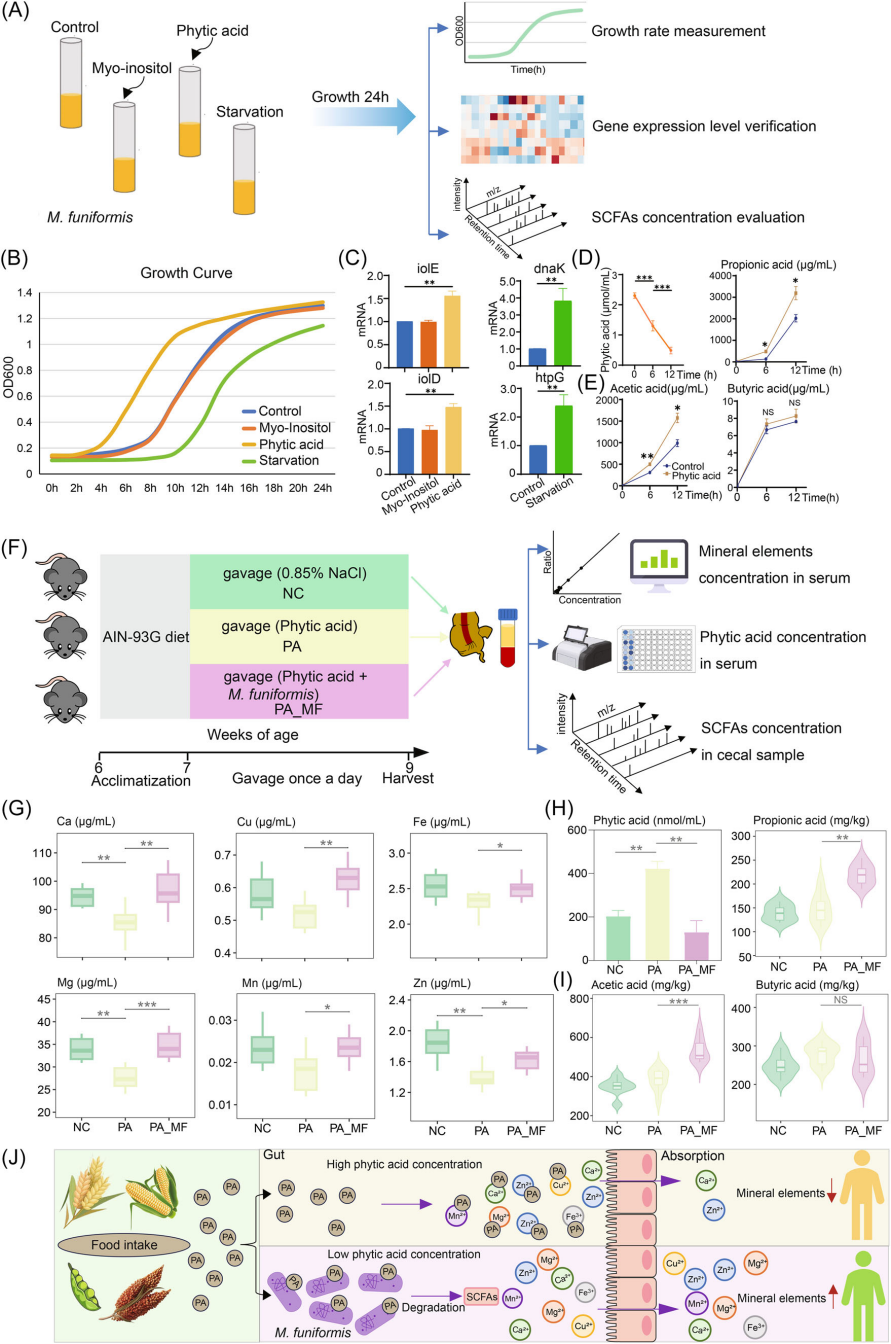
*M. funiformis* improve mineral absorption through exogenous phytic acid degradation. (A) The experimental design of in vitro *M. funiformis* phytic acid conversion. (B) The growth curve of *M. funiformis* at different culture conditions. (C) Bar plot showing the expression level of *iolE*, *iolD*, *dnaK* and *htpG* of *M. funiformis* in different conditions. (D) Line chart showing the phytic acid concentration in phytic acid group at different growth phases. (E) Line chart showing the acetic acid, propionic acid and butyric acid concentration in phytic acid group at different growth phases. (F) The experimental design of in vivo *M. funiformis* phytic acid conversion (*n* = 8). (G) Boxplot showing the serum mineral elements concentration in the different treatment groups. (H) Bar plot showing the phytic acid concentration in the different treatment groups. (I) Violin plot showing the acetic acid, propionic acid and butyric acid concentration in the different treatment groups. (J) Overview of the effect of phytic acid on mineral absorption and degradation by *M. funiformis* in gut. NC, mice treated with 0.85% NaCl; PA, mice treated with phytic acid; PA_MF, mice treated with phytic acid and *M. funiformis*. *p < 0.05, **p < 0.01, ***p < 0.001, NS, not significant. Ca, calcium; Cu, copper; Fe, iron; Mg, magnesium; Mn, manganese; SCFA, short‐chain fatty acids; Zn, zinc.

Given the high expression of stress and myo‐inositol‐related genes, and the activation of inositol phosphate metabolism pathways in *M. funiformis*, we conducted a series of experiments to explore its potential mechanisms. We cultured *M. funiformis* under various conditions: high concentrations of myo‐inositol, high concentrations of phytic acid (inositol hexakisphosphate, IP6), and nutrient‐deficient environments. After selecting a single colony from the Columbia Blood Agar (CBA) plate (Figure S6A), firstly, we measured the growth of *M. funiformis* under the multiple conditions, further quantify the differences in metabolism at the molecular level. Next, we measured the concentration of phytic acid in phytic acid treatment group to explore phytic acid degradation ability of *M. funiformis*. Finally, we aimed to further explore the metabolic products of *M. funiformis* in degradation phytic acid, in conjunction with previous studies [38, 39], we assessed the SCFAs content in the culture supernatant (Figure 7A).

The growth curve results showed that *M. funiformis* grew faster in the presence of phytic acid and exhibited higher expression levels of the gene *iolD* compared to other groups. In contrast, the addition of myo‐inositol had minimal effect on *M. funiformis* growth rate (Figure 7B,C), suggesting that the elevated expression of myo‐inositol related genes observed in our study may be due to *M. funiformis* metabolizing phytic acid. Additionally, we observed significantly reduced bacterial growth under nutrient‐deficient conditions, which was accompanied by significant upregulation of stress‐related genes (Figure 7C). Moreover, we also observed that the significantly decrease of phytic acid concentration in the culture supernatant of the stationary phase compared with the logarithmic phase (Figure 7D). For a better understanding of the phytic acid metabolism of *M. funiformis*, we next evaluated the SCFA's concentration in the culture supernatant. As expected, we found that in the stationary phase of phytic acid group, acetic acid and propionic acid levels, were significantly higher compared with the logarithmic phase, while the level of butyric acid has not shown great change, supporting that *M. funiformis* was able to degrade phytic acid into acetic and propionic acids (Figure 7E). We also performed a phytic acid metabolized strain (*Escherichia coli* K‐12 [40]) as positive control to evaluate the phytic acid metabolism performance of *M. funiformis* (Figure S6B,C).

To better understand the function of *M. funiformis* in the gut microbiota, we subsequently studied in vivo. After 1 week acclimatization, the 7‐week‐old C57BL/6J mice were randomly assigned into three groups (control, phytic acid, phytic acid + *M. funiformis*) and were gavaged daily for 2 weeks. Since previous studies reported that the diets high in phytic acid could inhibit the mineral uptake and absorption in the gastrointestinal tract [41], we collected the serum samples and evaluated the mineral elements concentration. To investigate the degradation of *M. funiformis* on phytic acid, we collected serum and cecal samples to determine the phytic acid and SCFAs content, respectively (Figure 7F).

The results showed that the minerals, especially Calcium (Ca), Magnesium (Mg), and Zinc (Zn), were significantly decreased in the phytic acid group, while the serum mineral levels in the *M. funiformis* addition group were significantly higher than those in the phytic acid group, which was in line with previous studies (Figure 7G) [38]. We found that the serum phytic acid level was lower in the *M. funiformis* group compared with phytic acid group, which demonstrated that the *M. funiformis* could degrade phytic acid in vivo (Figure 7H). Moreover, we found acetic acid and propionic acid levels were significantly higher in *M. funiformis* group than phytic acid and control group, however, the level of butyric acid has not shown great change, the results were in line with our in vitro findings (Figure 7I). Taken together, these results strongly suggest that *M. funiformis* could degrade phytic acid and produce acetic acid and propionic acid, indicating that *M. funiformis* have the potential to improve malnutrition resulting from excessive intake of foods high in phytic acid (Figure 7J).

## DISCUSSION

In this study, we applied a single‐microbe RNA sequencing method to dissect the functional redundancy and microbial heterogeneity at single microbe resolution in the human gut microbiome of three healthy donors representing enterotypes ET‐P, ET‐B, and ET‐F. We profiled nine samples from the three different enterotype donors at three time points intraday, and obtained functional heterogeneity between individuals with the inter‐ or intra‐species. In a previous study, we performed high‐throughput single‐microbe RNA sequencing of the human gut microbiome and was able to measure inter‐ and intra‐species adaptive strategy heterogeneity [10]. Building on this foundation, the current study focused on exploring species‐specific functional heterogeneity and the dynamic activity alterations in the gut microbe community function among humans with different enterotypes.

It is well‐established that different species in the human gut exhibit distinct functions [42]. Our results demonstrate that single‐microbe RNA sequencing effectively captures functional diversity based on single‐microbe transcriptional characteristics. Given that several species are present in different donors, we further investigated whether the metabolic functions of the same bacterial species vary among individuals. For example, *M. funiformis* was more active in amino acid metabolism in the ET‐P donor, whereas in the ET‐F donor, it was more active in lipid metabolism (Figure 4D). This suggests that the metabolic functions of the same bacterial species can change under different host conditions, such as diet or individual genetics [43]. Functional redundancy is a common phenomenon in the human gut microbiome [44]. Through single‐microbe RNA sequencing, we comprehensively dissected the functional redundancy and complementarity patterns in the human gut microbiome. For example, in the ET‐P donor, both *P. copri* (from the phylum Bacteroidetes) and *M. funiformis* (from the phylum Firmicutes) exhibited high carbohydrate metabolism activity (Figures 3, 4). The coexistence of microorganisms with similar roles can benefit the host, as other microbes can compensate for the loss of a beneficial strain by providing the same function. Overall, the diversity of microbial functions and functional redundancy appear to be crucial for maintaining resilience [43].

In addition, using single‐microbe RNA sequencing technology, we captured the diurnal dynamic activities of the human microbiome at single‐microbe resolution for the first time. Previous studies on the impact of long‐ or short‐term dietary changes on gut microbiota have primarily focused on the changes in the proportions of different microbial species [39, 45]. The traditional metagenomics only provides the relative abundance in the sample. However, the behavior and biological effects of a microbial community are determined not only by its species composition and diversity but also by the cell states that occur within each microbe. By analyzing fecal samples at three different timepoints during the day, we gained new insights into the dynamic patterns of the human gut microbiome. For example, in the ET‐F donor, the abundance of *M. funiformis* did not significantly change across the three time points (Figure S7). However, at the single‐microbe level, the carbohydrate metabolism activity of *M. funiformis* exhibited strong dynamic alterations, which is likely a response to food intake or host physiological shifts [46].

Furthermore, within the same species, *M. funiformis* (a species characteristic of individuals of Asian ethnicity), we identified various subpopulations displaying differential gene expression related to metabolic, stress‐response, or growth‐related pathways in the distinct colon ecosystems of different donors. Notably, we discovered that the inositol phosphate catabolism pathway was significantly activated in a sub‐population of *M. funiformis*, which suggested was related with the degradation of phytic acid (inositol hexakisphosphate, IP6). Phytic acid, a storage form of phosphorus, is a key antinutrient factor in plant‐based diets [47]. It has been reported that dietary phytic acid intake is higher in Asian countries compared to Western developed countries due to its accumulation in cereal grains, nuts, and legume seeds which constitute a greater part of the Asian diet [48]. In our study, we found that phytic acid supplementation significantly enhances the growth rate of *M. funiformis*, and the animal studies suggesting that *M. funiformis* could function as a probiotic by improving the bioavailability of calcium, magnesium, and zinc in human gut. Compared to previous high‐throughput methods for screening specific functional species or strains [39, 49], our current approach offers new insights into exploring species with key function specializations.

The present study has some limitations. For the sample collection, the sample size in this study is relatively small, with only one individual per enterotype. In future studies, we should collect samples before meals for control and include a larger number of donors across different enterotypes. For the experimental design, we only choose a phytic acid metabolized bacteria as positive control without a negative control, the mineral element was only conducted on serum samples and the *M. funiformis* we used to couduct a series of experiments was not directly isolated from the certain donor. Since the diurnal dynamic of the human gut microbiome is influenced by various factors (e.g., diet and endocrine), so we performed analysis on the time‐specific species in vivo without in vitro.

## CONCLUSION

In summary, we systematically investigated the human gut microbiome's species functional specialization and redundancy, the diurnal dynamic activities and the microbe state transition heterogeneous, at a higher resolution than previous observations. We were able to better resolve the microbe cellular state transitions of the human gut microbiome based on single‐microbe RNA sequencing in distinct colon ecosystems greatly improving our understanding of the species‐function heterogeneity among individuals. Furthermore, our study demonstrated that the single‐microbe RNA sequencing and analytic framework was an efficient strategy to identify keystone species with specialized metabolic function of biological and clinical importance in the human gut microbiome. These new findings and developed novel methods could potentially contribute to precision microbiome diagnosis and treatment strategies, thereby improving human health outcomes.

## MATERIALS AND METHODS

### Human fecal sample collection

Nine fecal samples were collected from three healthy donors (male, age range 20–30) with distinct enterotypes at three different time points: Time point 1 (TP1) (6–9 ante meridiem (a.m.), after breakfast), Time point 2 (TP2) (11 a.m.–1 post meridiem (p.m.), after lunch), and Time point 3 (TP3) (6–9 p.m., after dinner). The dietary of the donors was based on their regular daily eating habits. The study protocol was approved by the Ethics Committee of the First Affiliated Hospital, Zhejiang University School of Medicine, China (2021IIT A0239), and all participants provided written informed consent. The samples were centrifuged twice at 4°C, 500 *g* for 3 min to remove impurities from digested food or host cells. The samples were also performed a shotgun metagenomic sequencing for identification and different enterotypes were clustered at genus level. The purified samples were then centrifuged at 4°C, 3900 *g* for 5 min to collect bacteria, which were subsequently used for single‐microbe RNA sequencing.

### Single‐microbe RNA sequencing of the human gut microbiome

#### Microbe fixation and permeabilization

To prevent degradation, human stool samples were directly resuspended and dispersed in 4% paraformaldehyde (100496, Sigma). The dispersed solutions were gently inverted overnight at 4°C to ensure complete fixation. The overnight‐fixed solutions were centrifuged at 200 *g* for 5 min to precipitate impurities in the stool samples. The supernatants were transferred and filtered through cell strainers (43‐50000‐98, pluriSelect) to improve the isolation of impurities. The filtered solutions were washed with ice‐cold phosphate buffer saline (PBS)‐RNase Inhibitor (RI) (1× PBS (10010072, Thermo Fisher) supplemented with 0.5 U/µL RI (N8080119, Thermo Fisher, United States) and resuspended in 0.04% Tween‐20 (655206, Sigma) in PBS. The microbes were mixed with a cell wall digestion mix (R20115124, M20 Genomics) and incubated at 37°C for 15 min. Immediately after the digestion, the microbes were resuspended in ice‐cold PBS‐RI to terminate the incubation. The microbes were washed with ice‐cold PBS‐RI and resuspended in ice‐cold 27.5 µL diethyl pyrocarbonate (DEPC) water (693520, Sigma), containing approximately 5 million bacteria for the following in situ reactions.

#### *In situ* reverse transcription and deoxyadenine (dA) tailing

In situ reactions were performed with VITApilote‐PFT1200 kit (R20114124, M20 Genomics). The microbes were mixed with 10 microliter (µL) 5× reverse transcription buffer, 5 µL 10 micromolar (µM) random primer, 2.5 µL 100 mM deoxynucleoside triphosphates (dNTPs), 2.5 µL RNase inhibitor, and 2.5 µL reverse transcriptase (50 U/µL). The reaction mixtures were incubated in a thermal cycler for 10 cycles of annealing, with the temperature gradually increasing from 8°C to 42°C, and then finishing with a 30‐min incubation. After reverse transcription, the microbes were washed with ice‐cold PBS‐(Tween‐20) T (0.05% Tween‐20 in 1× PBS). The washings were repeated for three times, and the microbes were subsequently resuspended in 39 µL DEPC water. The microbes were mixed with 5 µL 2.5 millimolar millimolar (mM) CoCl₂, 5 µL 10× Terminal deoxynucleotidyl transferase (TdT) buffer, 0.5 µL 100 mM deoxyadenosine triphosphate (dATP), and 0.5 µL TdT enzyme. The microbes were incubated at 37°C for 30 min to perform in situ dA tailing. After dA tailing, the microbes were washed with ice‐cold PBS‐T. The washings were repeated for two times and the microbes were subsequently resuspended in 200 µL PBS‐T.

#### Single‐microbe droplet encapsulation

Single‐microbe droplets were prepared following our previous developed protocols [10], and utilizing the VITApilote‐PFT1200 kit. Briefly, the microbes were counted and adjusted to an optimal cell density using a density gradient solution. The microbes, 2× DNA extension reaction mix, and barcoded hydrogel beads (Table S4) were loaded into the corresponding inlets in the microfluidic chip. The single‐microbe droplets were subsequently generated by the microfluidic platform VITAcruizer DP400. After encapsulation, the droplets were incubated at 37°C for 1 h, 50°C for 30 min, 60°C for 30 min, and 75°C for 20 min.

#### cDNA enrichment

The droplets were mixed with Perfluorooctanol (370533, Sigma) buffer to isolate the aqueous phase of the sample from the oil phase. The aqueous phase mixtures were purified with DNA cleanup magnetic beads (A63882, Beckman). After purification, quantitative polymerase chain reaction (qPCR) was conducted to identify the optimal cycle numbers for complementary DNA (cDNA) enrichment. This process pinpointed the early exponential amplification phase corresponding to these cycles. PCR amplifications were performed with the primer sets described in Table S5. The PCR products were purified with magnetic beads and quantified using Qubit 3.0 (Q33216, Thermo Fisher) and qualified with the DNA Fragment Analyzer (Qsep100, Bioptic).

#### Library preparation and sequencing

The DNA Library Prep Kit for Illumina V3 (ND607‐03/04, Vazyme) was utilized for library construction. Qualified cDNAs were mixed with end‐repair enzymes, end‐repair buffer, and nuclease‐free water to perform end‐repair and adenylation. The reaction mixtures were incubated at 30°C for 30 min and then heat‐inactivated at 65°C for 30 min. The heat‐inactivated mixtures were mixed with working adaptors and ligation enzymes and incubated at 20°C for 15 min. After incubation, DNA purifications and selections were performed with magnetic beads. The libraries were amplified via PCR and purified with magnetic beads. The purified cDNA libraries were subsequently quantified and qualified before sequencing on the NovaSeq. 6000 platform with the S4 Reagent Kit, generating paired‐end reads of 150 base pairs (bp).

### Human gut single‐microbe RNA sequencing data analysis

#### Data quality control, taxonomic annotation and gene expression level quantification

We first filtered the low‐quality barcodes and annotated the species of each barcode by our previously developed taxonomic annotation pipeline Microbe‐Annotation (MIC‐Anno) [10]. To examine functional heterogeneity within species, we focused on abundant bacterial species, defined as those represented by more than 100 total barcodes. We began by trimming primer sequences and additional bases generated during the dA‐tailing step from the raw paired‐end sequencing data. From the paired‐end reads, we extracted the 8 bp unique molecular identifier (UMI) and 20 bp cell‐specific barcode from the R1 end sequencing file and merged them, accepting barcodes with a Hamming distance of 2 bp or less. The R2 end file was used to generate the bacterial gene expression matrix using STAR (v2.7.10a) [50], featureCounts (v2.0.3) [51] and umi_tools (v1.1.2) [52] with appropriate parameters and the whole UHGG (v2.0.1) [21] gut microbiome genome as the reference. Only uniquely mapped reads were retained to count UMIs for each barcode.

#### Dimensionality reduction clustering and functional marker gene identification

The single‐microbe gene expression matrix was imported into Seurat (version 4.1.3) [53] for the downstream analysis. Microbes with nCount_RNA > 30 and nFeature_RNA > 15 were retained for further study. We define species with more than 100 barcodes as the “core species.” Dimensionality reduction was performed using principal component analysis (PCA) and clustering was performed using uniform manifold approximation and projection (UMAP). Clusters were identified with the “FindClusters” function (resolution = 0.5) in Seurat. For the clustering analysis in single microbe species, microbes from the species “*P. copri*” and “*B. dorei*” in the ET‐B donor, and “*M. funiformis*” in the ET‐P and ET‐F donors were extracted and subclusters were identified using the “FindClusters” function (resolution = 0.5). Marker genes were determined with the “FindAllMarkers” function (adjusted *p*‐value < 0.05) in Seurat, using the two‐sided Wilcoxon rank‐sum test with Bonferroni correction to identify unique transcriptional differences among microbes. Analysis of similarities (ANOSIM) was used to determine whether there are statistically significant differences between different time points in each species.

#### MIC‐Metabolism

MIC‐Metabolism was developed to quantify single‐microbe metabolic activity using single‐microbe RNA sequencing data. Its primary function is to assess metabolic pathway gene set activity in individual microbes across different species. To annotate gene functions in gut microbiome species, we used UHGG (v2.0.1) [21] to obtain KEGG orthology (KO) and queried KEGG pathways using the KEGGREST package (v1.38.0) to compile a list of metabolic gene sets.

The quantification method in MIC‐Metabolism calculates the ranked‐based MES for each species through the following three steps: (1) Raw MES generation: In this study, we used a ranked‐based method ssGSEA [54] to calculate the metabolic gene set enrichment score of each microbe. MIC‐Metabolism also supports different methods: ssGSEA, AUCell [55], VISION [56], and GSVA [57], of which ssGSEA is the default method. The input data is an expression matrix, in which the values are gene‐summarized counts. (2) Permutation‐based activity inference: We performed a permutation analysis on genes within each specific pathway 1000 times, resulting in 1000 random enrichment scores. The calculated raw MES in Step 1 was then compared against the random score sets. For example, if it ranked within the top 10, corresponding to a pathway *p*‐value < 0.01, the pathway was considered “significantly activated” in the species. (3) MES normalization: The normalization method is based on the permutation analysis results, where the *x*‐axis is the ranking of raw MES, and the *y*‐axis is the MES of specific pathway; we used the function “scale” in R to get the z‐score as the final normalized MES, if the MES is greater than 0, the pathway was considered as “activated,” while if the MES is lower than 0, the pathway was defined as “inactive.” MES correlation analysis was used to investigate pathway activity co‐occurrence patterns between species based on Spearman correlation analysis.

#### Pseudotime and gene enrichment analysis

After the clustering analysis, the microbe species “*M. funiformis*” from ET‐P and ET‐F donors was extracted for pseudotime analysis. We used the Monocle 2 package (version 2.26.0) [58] to explore the microbe cellular state transitions in distinct colon ecosystem. Differential expressed gene analysis was conducted with the “differentialGeneTest” function, considering genes with a *q*‐value < 0.01 as differentially expressed genes (DEGs). These DEGs were sorted and imported into the cell data set using the “setOrderingFilter” function. The pseudotime trajectory was constructed with the “DDRTree” algorithm using default parameters and visualized with the “plot_cell_trajectory” function. The dynamic expression changes of the DEGs were visualized using the “plot_pseudotime_heatmap” function. KEGG enrichment analysis was performed using the “enricher” function of the clusterProfiler package (version 4.6.0). All analyses were conducted in R (version 4.2.2).

### Growth rate measurement of *M. funiformis* in multiple conditions

The bacteria used for the growth curve experiment was *Megamonas funiformis* DSM 19343 (*M. funiformis*) bought from the German Collection of Microorganisms and Cell Cluture (DSMZ). The *M. funiformis* DSM 19343 is an anaerobe, gram‐negative, rod‐shaped bacterium that was isolated from human faeces in Japan [59]. *M. funiformis* was first cultured overnight on CBA solid culture medium at 37°C, in an anaerobic bag (Figure S6A). A single colony was then selected from the CBA plate and inoculated into Chopped Meat Carbohydrate Broth (CMC) liquid culture medium (KDM150, MingZhouBio) at 37°C in an anaerobic bag without shaking for further experiments. When the liquid culture *M. funiformis* reaching OD_600_ ~ 0.6, equal volumes (100 µL) of bacterial suspension were inoculated into different culture conditions (including those supplemented with 50 μg/mL myo‐inositol (Myo‐Inositol group), 50 μg/mL phytic acid (Phytic acid group), and 70% nutrient‐deficient medium (Starvation group), and no treatment was applied except for the addition of *M. funiformis* (Control group), all the volume was 10 mL). For each group, five replicate were set. Optical density (OD) at 600 nm was measured every 2 h after inoculation to construct bacterial growth curves. We then collected the culture supernatant of phytic acid group at 0 h, 6 h, and 12 h for phytic acid measurement and the culture supernatant of control and phytic acid group at 0 h, 6 h, and 12 h for SCFAs measurement.

### A phytic acid metabolized strain (*Escherichia coli* K‐12) as positive control

*E. coli* K‐12 (ATCC 25404) was first cultured overnight on Luria‐Bertani Broth (LB) solid culture medium at 37°C. A single colony was then selected from the LB plate and inoculated into LB liquid culture medium (KDM159, MingZhouBio) at 37°C without shaking for further experiments. When the liquid culture *E. coli K‐12* reaching OD_600_ ~ 0.6, equal volumes (100 µL) of bacterial suspension were inoculated into culture conditions with 50 μg/mL phytic acid (Phytic acid group) and no treatment was applied except for the addition of *E. coli* (Control group), all the volume was 10 mL. We then collected the culture supernatant of phytic acid group at 0 h, 6 h, and 12 h for phytic acid measurement and the culture supernatant of control and phytic acid group at 0 h, 6 h, and 12 h for SCFAs measurement.

### Real‐time qPCR analysis

Total RNA was extracted from *M. funiformis* at different culture conditions by FreeZol Reagent (R711‐01, Vazyme). cDNA was synthesized using the HiScript IV RT SuperMix for qPCR (+gDNA wiper) (R423‐01, Vazyme). Quantitative real‐time PCR (RT‐qPCR) was performed on the CFX96 system (Applied Biosystems) using ChamQ Universal SYBR qPCR master mix (Q711‐02, Vazyme). Gene expression levels were normalized to those of 16s ribosomal RNA (rRNA). Primers used in this study are shown in Table S6. The gene expression levels among and between groups were statistically evaluated using Analysis of Variance (ANOVA).

### Animal studies

Six‐week‐old male C57BL/6J mice were purchased from the First Affiliated Hospital, Zhejiang University School of Medicine, and were randomly divided into four mice per cage, with free access to food and water under a strict 12 h light cycle. Acclimatization time was 1 week. Mice were fed AIN‐93G diet (purchased from Shuangshi experimental animal feed), and randomly assigned into three groups (each group with 8 mice). To test the in vivo degradation of phytic acid by *M. funiformis*, mice were gavaged with 0.2 mL 0.2 mg g^−1^ body weight of phytic acid (phytic acid group), or 0.2 mL 0.85% NaCl (Control group), or 0.2 mL 0.2 mg g^−1^ body weight of phytic acid and 1*10^9^ colony‐forming units (c.f.u.s) of *M. funiformis* (phytic acid + *M. funiformis* group) every day for 2 weeks. Bacterial suspension was prepared in 0.85% NaCl. In Week 3, mice were fasted for 12 h before collect blood and colon contents. Blood samples were placed in plastic tubes for at least 3 h and then centrifuged at 4000 rpm for 10 min at 4°C. The serum was then collected to be used for mineral content determination and phytic acid concentration determination. After blood sampling, the cecal samples were taken and used for SCFAs evaluation. The study was reviewed and approved by the Animal Experimentation Ethics Committee of the First Affiliated Hospital College of Medicine, Zhejiang University, with approval number 20241356. All methods involving animals were carried out in accordance with the ARRIVE (Animal Research: Reporting of In Vivo Experiments) guidelines.

### Measurement of minerals

The levels of copper (Cu), iron (Fe), zinc (Zn), manganese (Mn), magnesium (Mg), and calcium (Ca) in mouse serum were evaluated using an inductively coupled plasma mass spectrometry (ICP‐MS NexION 1000G, PerkinElmer) according to previous study [60]. Add 100 μL serum into a Polytetrafluoroethylene (PTFE) digestion vessel and add 0.5 mL 68% HNO_3_, and add reagents to another PTFE digestion vessel as blank control, then cover the digestion vessel tightly after bubbles have completely disappeared. Place the digestion vessel in a microwave digestion system and start digestion according to the digestion protocol. Once cooled, transfer the digested solution to a 10 mL volumetric flask and dilute to 5 mL using ddH_2_O. After filtering the diluted sample through a 0.22 μm membrane, using ICP‐MS for detection. The elements concentration in the serum could be calculated as following formula:

$$\text{Element concentration }(\mu g/\text{mL})=\frac{C\times V_{\mathrm{final}}}{V_{\mathrm{sample}}},$$

*C*: the concentration of solution detected by ICP‐MS; *V*_final_: diluted volume, in this method is 5 mL; *V*_sample_: sample volume used in the detection, in this method is 100 μL.

### Measurement of phytic acid

The level of phytic acid in serum or culture supernatant was determined using phytic acid content assay kit (BC5845, Solarbio,) according to the manufacturer's instructions. Step1: Add 100 μL serum or culture supernatant into 1 mL extraction solution I, and shake at 25°C for 2 h. Then centrifuge at 10,000 *g* for 10 min at 4°C, and collect 0.8 mL of the supernatant. Step 2: Slowly add 0.15 mL extraction solution II, gently mix, after centrifugation at 10,000 *g* for 10 min at 4°C, collect 120 μL supernatant. Step 3: Add 50 μL reagent II, incubating in water bath at 37°C for 30 min, then add 50 μL working solution and stand at 25°C for 10 min, measure at OD_700_ (*A*_measure_). Taken 120 μL supernatant from Step 2, add 50 μL reagent I instead, and follow the same procedure as step 3, measure at OD_700_ (*A*_control_). Taken 120 μL 250 nmol/mL standard phytic acid solution and follow the same procedure as step 3, measure at OD_700_ (*A*_standard_). Taken 120 μL reagent I and follow the same procedure as step 3, measure at OD_700_ (*A*_blank_). Each serum sample requires a corresponding control tube. The concentration of phytic acid in the serum or culture supernatant could be calculated as the following formulas:

$$\Delta A_{\mathrm{measure}}=A_{\mathrm{measure}}-A_{\mathrm{control}},$$

$$\Delta A_{\mathrm{standard}}=A_{\mathrm{standard}}-A_{\mathrm{blank}},$$

$$\text{Phytic acid }(\text{nmol}/\text{mL})=\frac{\;∆A_{\mathrm{measure}}\times C_{\mathrm{standard}}\times (V_{\mathrm{supernatant}}+V_{\mathrm{solution}\;\mathrm{II}})\times (V_{\mathrm{solution}\;I}+V_{\mathrm{total}})}{∆A_{\mathrm{standard}}\times V_{\mathrm{total}}\times V_{\mathrm{supernatant}}},$$

*C*_standard_: the concentration of standard phytic acid, in this kit is 250 nmol/mL; *V*_supernatant_: the volume of supernatant added in step 2, in this method is 120 μL; *V*_solution II_: the volume of extraction solution II in step 2, in this method is 0.15 mL; *V*_solution I_: the volume of extraction solution I in step 1, in this method is 1 mL; *V*_total_: the volume of total liquid after adding working solution, in this method is 0.1 mL. The Mann–Whitney *U* test was used to compare continuous variables.

### Measurement of SCFAs

The SCFAs concentration in cecal samples or culture supernatant was measured using the gas chromatography‐mass spectrometry (GC‐MS 7890A‐5975C, Agilent). For the cecal samples, the following steps were applied: 1. Add 1 mL pure water in 25 mg cecal sample and vortex 10 s; 2. Add steel beads and process with a 40 Hz grinder for 4 min, followed by ultrasonic treatment in an ice water bath for 5 min (repeat three times); 3. Centrifuge the sample at 4°C 5000 rpm for 20 min; 4. Transfer 0.8 mL supernatant into a new 2 mL Eppendorf (EP) tube; 5. Add 0.1 mL 50% H_2_SO_4_ and 0.8 mL internal standard solution (200 μg/mL 2‐Ethybutyric acid, using methyltert‐butylether (MTBE) as the solvent) (214353, Fisher), vortex for 10 s, oscillate for 10 min, and sonicate for 10 min in an ice water bath; 6. Centrifuge at 4°C 10,000 rpm for 15 min, stand at −20°C for 30 min and remove 100 μL supernatant for GC‐MS analysis. For the culture supernatant samples, the following steps were applied: 1. Add 0.05 mL 50% H_2_SO_4_ and 0.2 mL internal standard solution in 100 μL sample, vortex for 30 s, oscillate for 10 min, and sonicate for 10 min in an ice water bath; 2. Centrifuge the sample at 4°C 10,000 rpm for 15 min; 3. Stand at −20°C for 30 min and remove 100 μL supernatant for GC‐MS analysis.

### Statistic methods

The two‐sided Wilcoxon rank‐sum test with Bonferroni correction was used to identify unique transcriptional differences among microbes. ANOSIM was used to determine whether there are statistically significant differences between different time points in each species. The gene expression levels among and between groups were statistically evaluated using ANOVA. MES correlation analysis was used to investigate pathway activity co‐occurrence patterns between species based on Spearman correlation analysis.

## AUTHOR CONTRIBUTIONS

**Yifei Shen**: Writing—original draft; supervision; writing—review and editing; project administration. **Wenxin Qu**: Writing—original draft. **Mengdi Song**: Formal analysis; writing—original draft. **Tianyu Zhang**: Data curation; writing—original draft. **Chang Liu**: Methodology; visualization. **Xiaofeng Shi**: Methodology; visualization. **Xinxin Xu**: Data curation. **Jingjing Jiang**: Data curation; Methodology. **Liguo Ding**: Methodology. **Fangyu Mo**: Validation. **Zheying Mao**: Validation. **Mingzhu Huang**: Formal analysis. **Ziye Xu**: Formal analysis. **Jiaye Chen**: Software. **Enhui Shen**: Software. **Jian Ruan**: Validation. **Jiong Liu**: Investigation. **Michael P. Timko**: Writing—review and editing; conceptualization. **Yu Chen**: Conceptualization. **Longjiang Fan**: Conceptualization. **Shufa Zheng**: Supervision; methodology; funding acquisition; writing—review and editing; project administration. **Yongcheng Wang**: Supervision; methodology; funding acquisition; writing—review and editing; project administration.

## CONFLICTS OF INTEREST STATEMENT

Yongcheng Wang is a cofounder, and Jiong Liu is a cofounder and employee of M20 Genomics. The other authors declare no competing interests. The result that *M. funiformis* metabolize phytic acid to produce acetic acid and propionic acid has been filed for patent protection by Zhejiang University.

## ETHICS STATEMENT

The study protocol was approved (No. 2021IIT A0239) by the Ethics Committee of the First Affiliated Hospital, Zhejiang University School of Medicine, China. All the participants provided written informed consent. The animal experimentation was approved (No. 20241356) by the Animal Experimentation Ethics Committee of the First Affiliated Hospital College of Medicine, Zhejiang University.

## Supporting information


**Figure S1.** The median gene number and species number in each sample among the three donors.
**Figure S2.** UMAP color by donors and expression level of *Desulfovibrio piger* marker gene.
**Figure S3.** Gene expression level of each species.
**Figure S4.** Correlation analysis of MES for the main species of ET‐P donor.
**Figure S5.** UMAP color by time points and donors of *Megamonas funiformis*, enrichment analysis of marker genes in cluster 3.
**Figure S6.** Colony morphology of *M. funiformis* on Columbia Blood Agar and experimental results of *Escherichia coli in vivo*.
**Figure S7.** The proportion of each species at different time points.

**Table S1.** The marker genes of each species.
**Table S2.** The marker genes of time point in main species.
**Table S3.** The marker genes of subpopulations of *P. copri*, *B. dorei* and *M. funiformis*.
**Table S4.** Unique barcoded primers targeted on the hydrogel beads.
**Table S5.** Primers used for RT and cDNAs amplification.
**Table S6.** The primer used in *M. funiformis* growth test.

## Data Availability

The data that support the findings of this study are openly available in Github at https://github.com/MIC-seq/MIC-seq-analysis-workflow. The single‐microbe RNA sequencing datasets produced in this study are available in Genome Sequence Archive under the BioProject accession code “PRJCA036556” (https://ngdc.cncb.ac.cn/bioproject/browse/PRJCA036556). The source data of the experiment is supplied in this paper. The code of MIC‐Metabolism pipeline and further analysis of single‐microbe RNA sequencing data has been presented on Github (https://github.com/MIC-seq/MIC-seq-analysis-workflow/tree/main/MIC-Metabolism). Supplementary materials (figures, tables, graphical abstract, slides, videos, Chinese translated version, and update materials) may be found in the online DOI or iMeta Science http://www.imeta.science/.
