# Supplementary material for: Single‐microbe RNA sequencing uncovers unexplored specialized metabolic functions of keystone species in the human gut
Source: Imeta. 2025 Apr 17;4(3):e70035. doi: 10.1002/imt2.70035 (PMC12130570; doi:10.1002/imt2.70035)
Supplement: Supplementary file 1 — Figure S1. The median gene number and species number in each sample among the three donors. Figure S2. UMAP color by donors and expression level of Desulfovibrio piger marker gene. Figure S3. Gene expression level of each species. Figure S4. Correlation analysis of MES for the main species of ET‐P donor. Figure S5. UMAP color by time points and donors of Megamonas funiformis, enrichment analysis of marker genes in cluster 3. Figure S6. Colony morphology of M. funiformis on Columbia Blood Agar and experimental results of Escherichia coli in vivo. Figure S7. The proportion of each species at different time points. [file IMT2-4-e70035-s001.docx]

**Supporting information to**

**Single-microbe RNA sequencing uncovers unexplored specialized metabolic functions of keystone species in the human gut**

**Running title:** Uncovering dynamic metabolic functions of human gut microbiome

Yifei Shen^1,2#*^, Wenxin Qu^1,2#^, Mengdi Song^1#^, Tianyu Zhang^1#^, Chang Liu^1,2^, Xiaofeng Shi^1^, Xinxin Xu^1^, Jingjing Jiang^1,2^, Liguo Ding^1^, Fangyu Mo^3^, Zheying Mao^1,2^, Mingzhu Huang^1,2^, Ziye Xu^1^, Jiaye Chen^1^, Enhui Shen^3^, Jian Ruan^4^, Jiong Liu^5^, Michael P. Timko^6^, Yu Chen^1,2^, Longjiang Fan^3^, Shufa Zheng^1,2*^, Yongcheng Wang^1*^

^1^ Department of Laboratory Medicine of The First Affiliated Hospital & Liangzhu Laboratory, Zhejiang University School of Medicine, Hangzhou, 310003, China.

^2^ Key Laboratory of Clinical In Vitro Diagnostic Techniques of Zhejiang Province, Hangzhou, 310003, China.

^3^ Institute of Bioinformatics, Zhejiang University, Hangzhou, 310058, China.

^4^ Department of Medical Oncology of The First Affiliated Hospital, Zhejiang University School of Medicine, Hangzhou, 310003, China.

^5^ M20 Genomics, Hangzhou, 310058, China.

^6^ Departments of Biology and Public Health Sciences, University of Virginia, Charlottesville, VA22904, USA.

^#^ These authors contributed equally: Yifei Shen, Wenxin Qu, Mengdi Song, Tianyu Zhang.

*Correspondence: [yongcheng@zju.edu.cn](mailto:yongcheng@zju.edu.cn) (Yongcheng Wang), zsfzheng@zju.edu.cn (Shufa Zheng), yifeishen@zju.edu.cn (Yifei Shen)


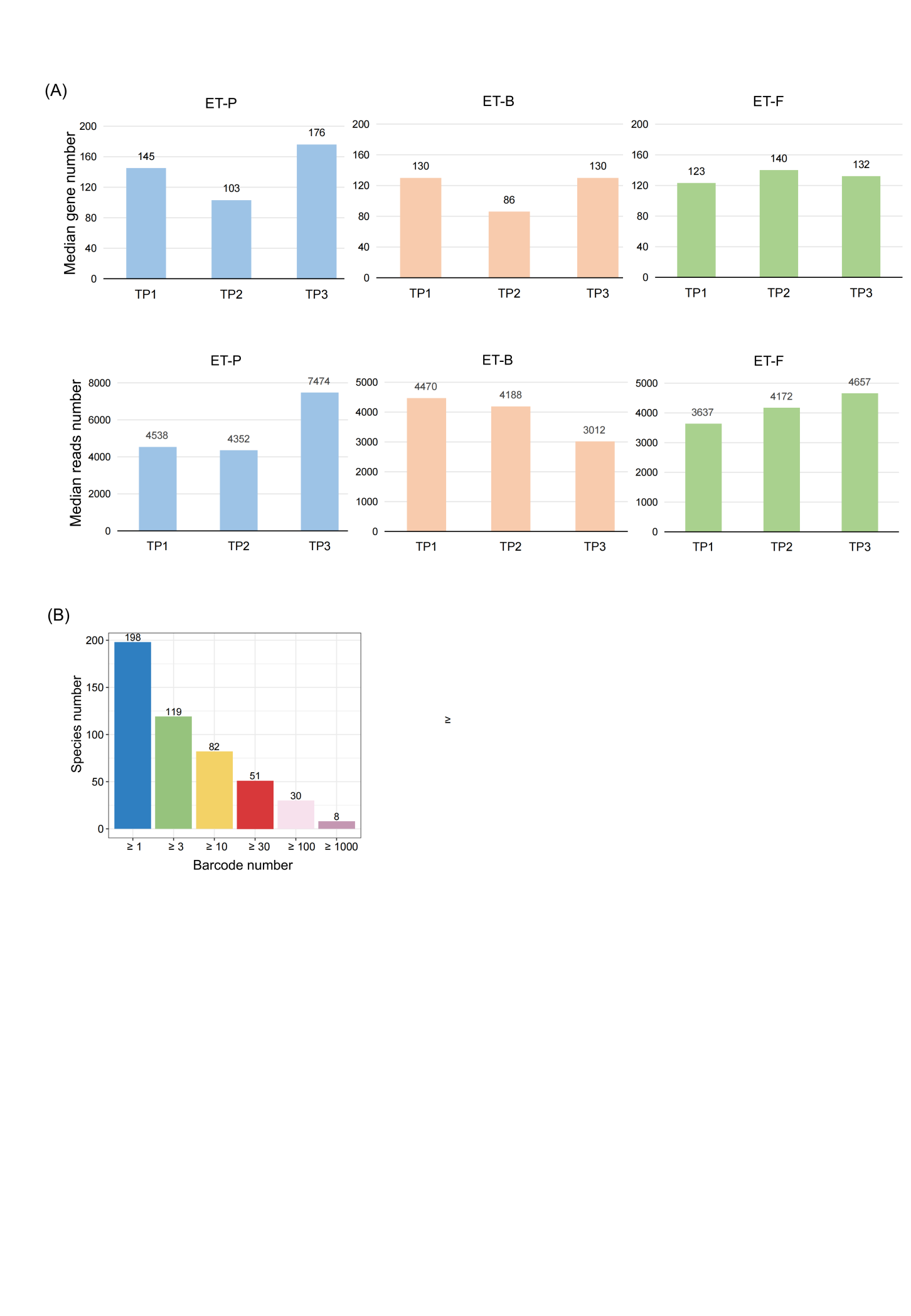


**Figure S1 The gene number and species number in each sample among the three donors.** (A) The gene number and reads number of each sample. (B) Species numbers of different cell number thresholds..

**
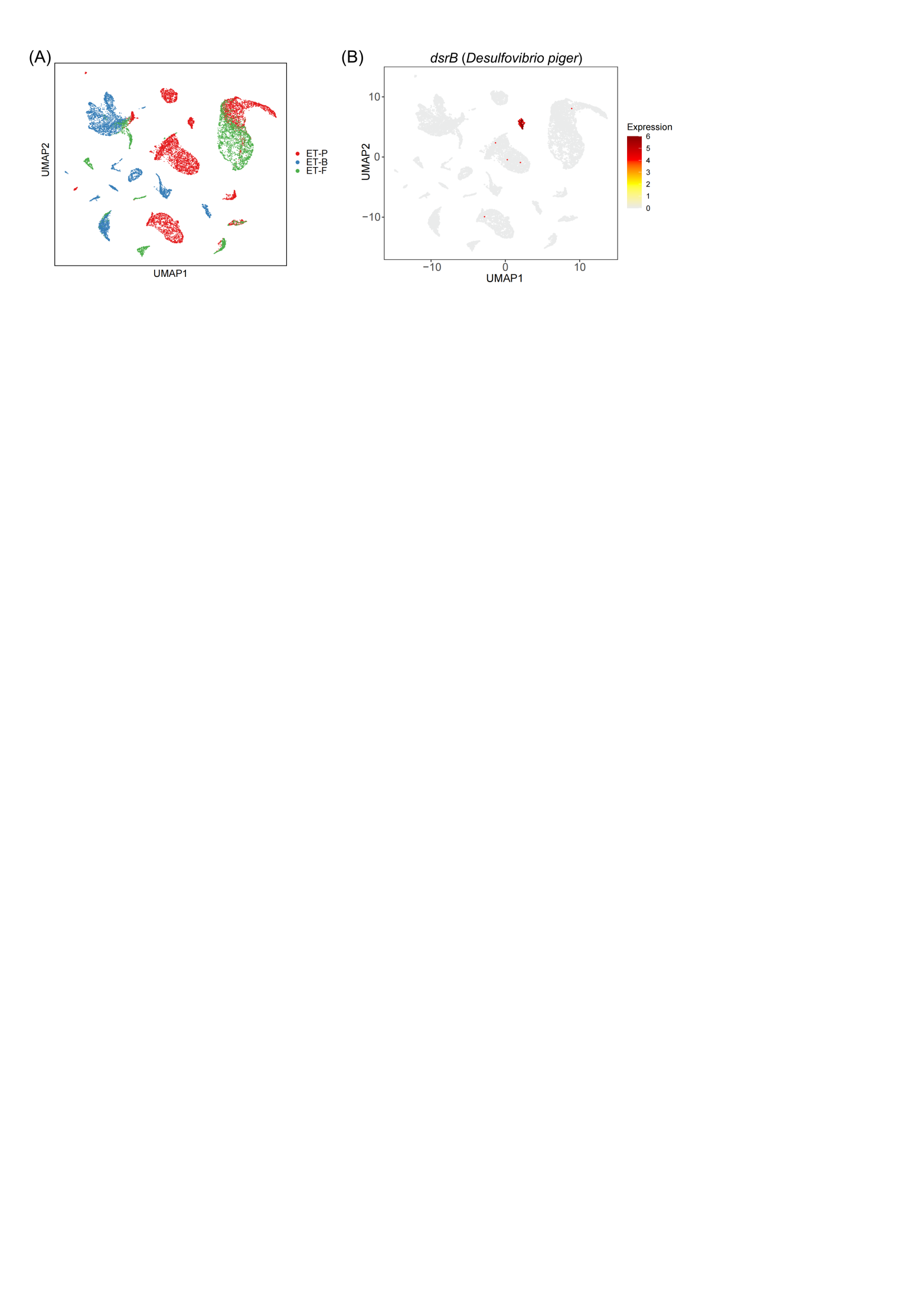
**

**Figure S2 UMAP color by donors and expression level of *Desulfovibrio piger* marker gene.** (A) UMAP color by different donors. (B) UMAP color by expression level of *D.piger* marker gene (*dsrB*).


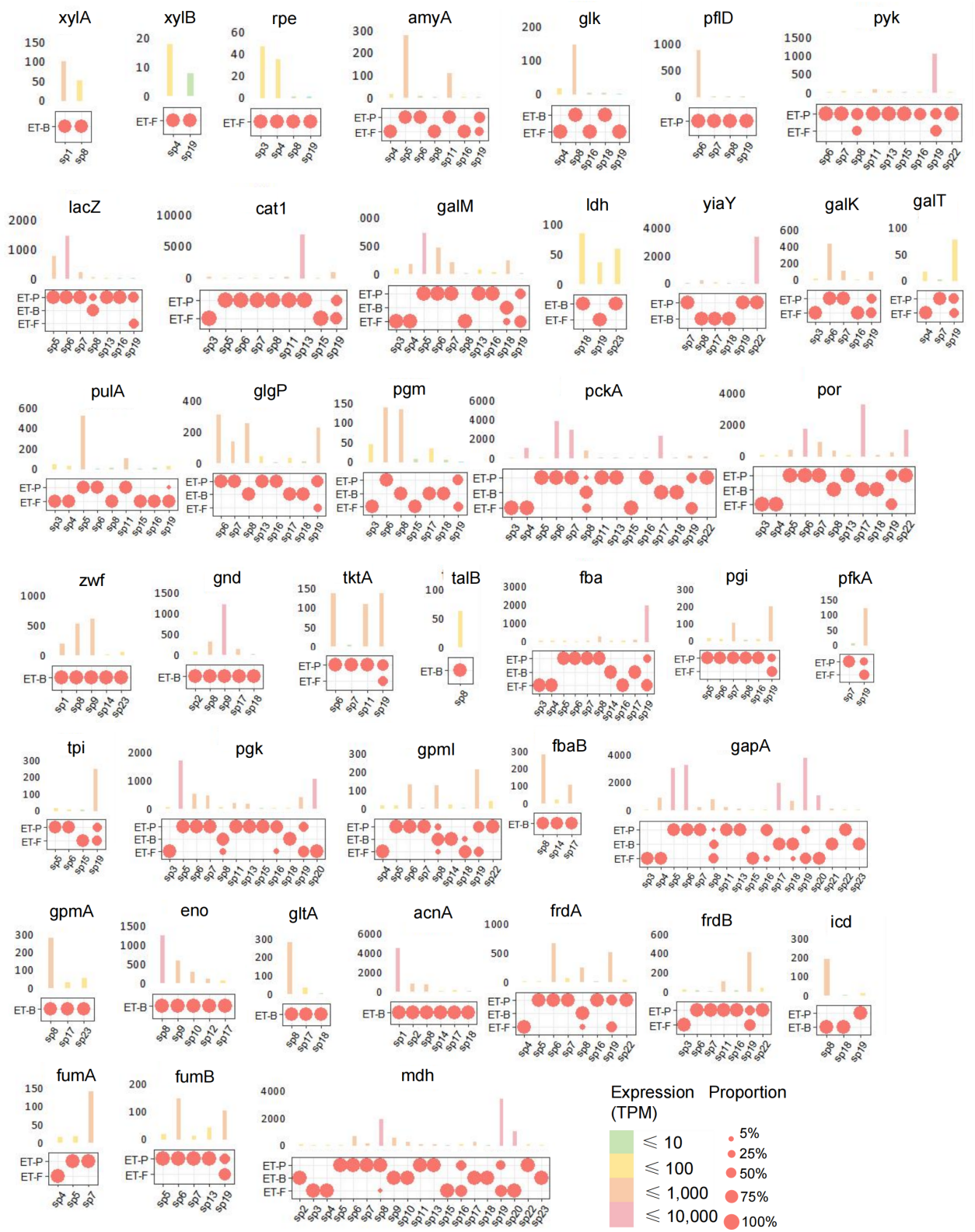


**Figure S3 Gene expression level of each species.** The bar plot showed the gene expression (TPM) of each species. The dot plot depicted the proportion of gene expression in different donors (e.g., the expression of *pulA* in *Prevotella hominis*, is contributed from ET-P donor). The sp1-23 was the same as Figure 3A.


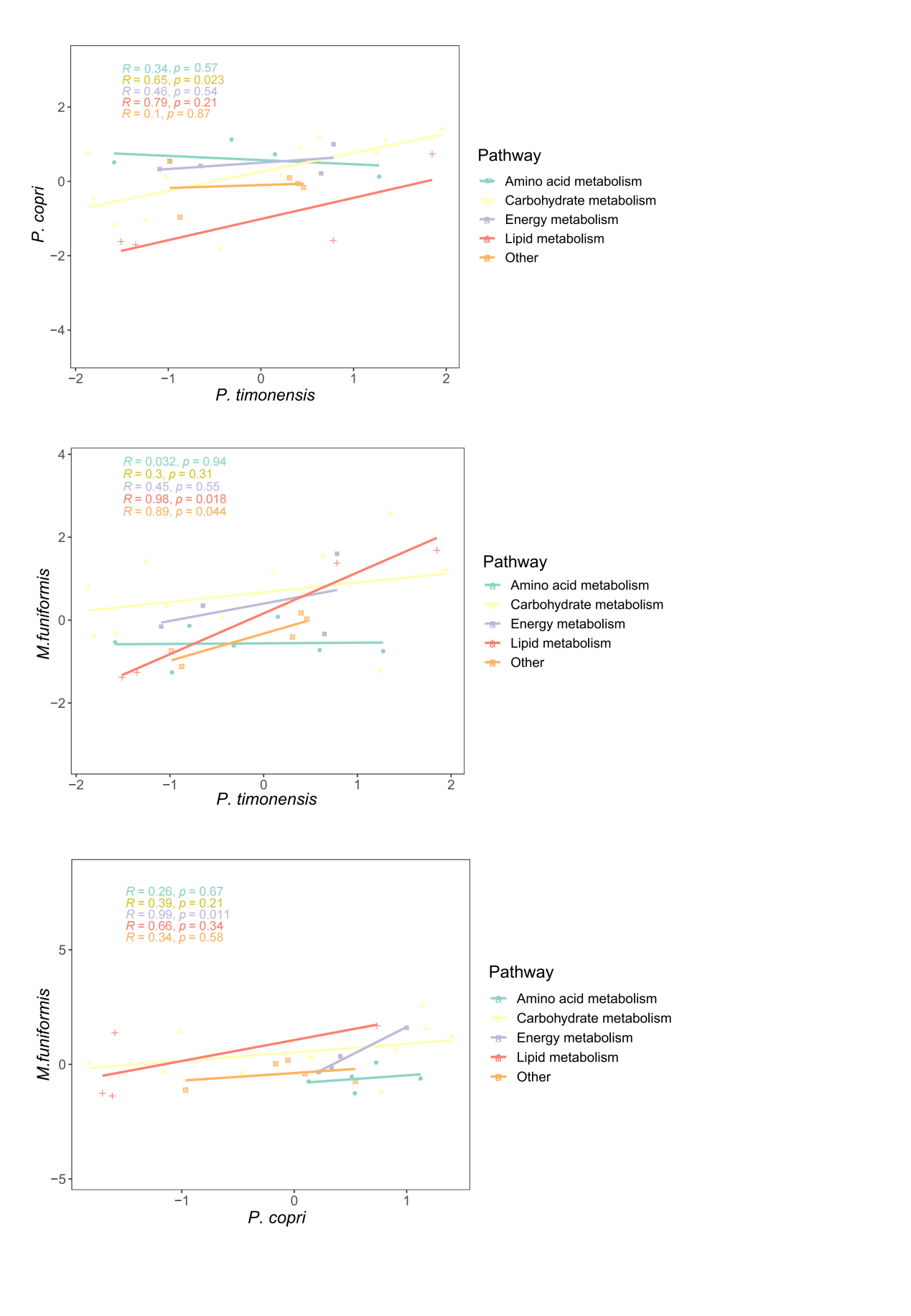


**Figure S4 Correlation analysis of MES for the main species of ET-P donor.**


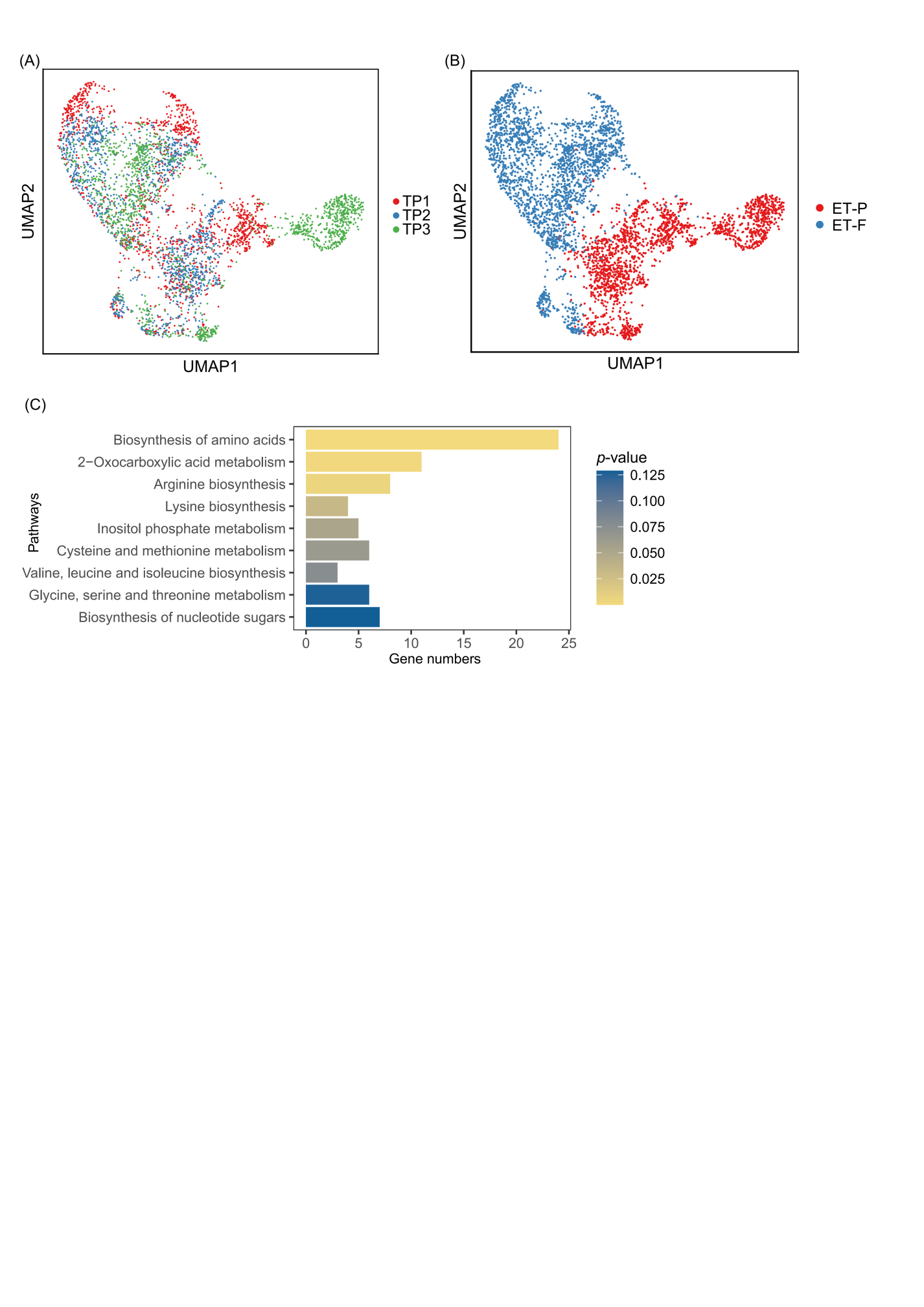


**Figure S5 UMAP color by time points and donors of *Megamonas funiformis*, enrichment analysis of marker genes in cluster 3.** (A) UMAP of *M. funiformis* color by different time points. (B) UMAP of *M. funiformis* color by donors. (C) The enrichment analysis of cluster 3 of *M.funiformis* marker genes.


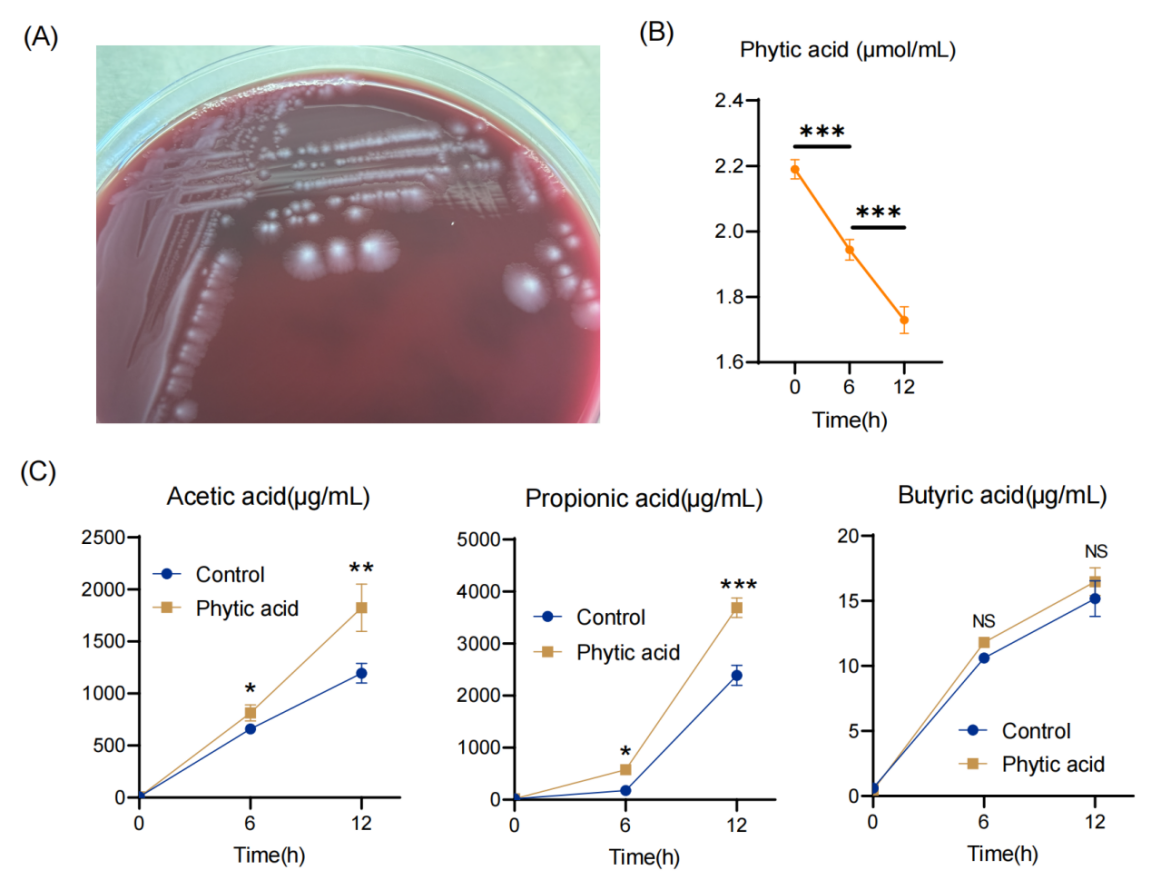


**Figure S6 Colony morphology of *M. funiformis* on Columbia Blood Agar and experimental results of *Escherichia coli in vivo****.* (A) Colony morphology of *M. funiformis* on Columbia Blood Agar. (B and C) The concentration of phytic acid (B), acetic acid, propionic acid and butyric acid (C) in the culture supernatant of *E.coli*.


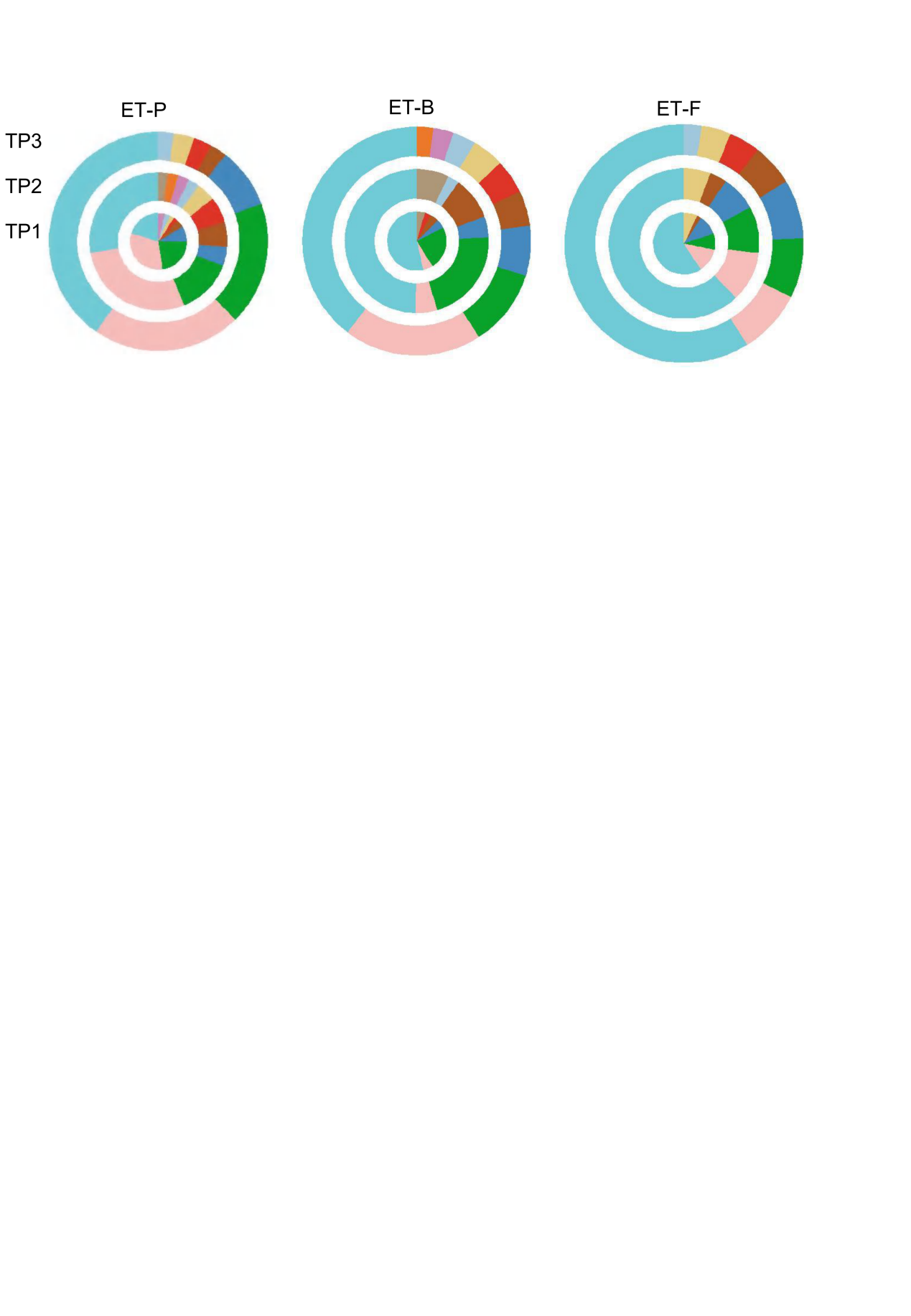


**Figure S7 The proportion of each species at different time points.** The species color was the same as Figure 5A.
